# Experimental and In Silico Comparative Study of Physicochemical Properties and Antimicrobial Activity of Carboxylate Ionic Liquids

**DOI:** 10.3390/molecules29153668

**Published:** 2024-08-02

**Authors:** Nikolett Cakó Bagány, Eleonora Čapelja, Strahinja Kovačević, Maja Karaman, Sanja Podunavac-Kuzmanović, Slobodan Gadžurić, Sanja Belić

**Affiliations:** 1 Faculty of Science, University of Novi Sad, Trg D. Obradovića 3, 21000 Novi Sad, Serbia; nikolet.cakobaganj@dh.uns.ac.rs (N.C.B.); eleonora.boskovic@dbe.uns.ac.rs (E.Č.); maja.karaman@dbe.uns.ac.rs (M.K.); sanja.belic@dh.uns.ac.rs (S.B.); 2 Faculty of Technology, University of Novi Sad, Bulevar Cara Lazara 1, 21000 Novi Sad, Serbia; strahko@uns.ac.rs (S.K.); sanya.podunavac@uns.ac.rs (S.P.-K.)

**Keywords:** ionic liquids, density, viscosity, electrical conductivity, antimicrobial activity, HCA

## Abstract

The COVID-19 pandemic highlighted the need to create and study new substances with improved lipophilicity and antimicrobial properties, such as ionic liquids (ILs), with easily tunable physicochemical properties. Most ILs possess strong antibacterial effects, but ILs containing the imidazolium cation are even more effective than the positive control. Thus, in this study, three ionic liquids with 1-butyl-3-methylimidazolium cation and various carboxylate anions (phenylacetate, benzoate, and 4-methoxyphenylacetate) were synthesized and fully characterized. The interactions between the cations and anions were discussed based on the experimental density, viscosity, and electrical conductivity. From the measured electrical conductivity and viscosity, the Walden plot is constructed and ionicity of the studied ILs is discussed. The similarities and dissimilarities among the studied ILs and their physicochemical properties are analyzed by applying the hierarchical cluster analysis and in silico calculated properties. The antimicrobial activity of the studied ionic liquids is tested on two bacterial (*E. coli* and *P. aeruginosa*) and three fungi (*P. verrucosum*, *A. flavus*, and *A. parasiticus*) strains, finding that they showed improved antimicrobial activity compared to the individual components.

## 1. Introduction

In the 21st century, serious infections caused by microorganisms resistant to commonly used antimicrobial agents have become a global healthcare problem. The outbreak of the COVID-19 pandemic reinforced the importance of the synthesis and characterization of new antimicrobial substances. Therefore, designing new compounds, such as ionic liquids (ILs), that show antibacterial activities is very important.

Ionic liquids are compounds composed of organic cations and organic or inorganic anions. Their physicochemical properties can easily be tuned by carefully choosing cations and anions [1,2,3,4]. ILs have a melting point below 100 °C and unique properties such as low flammability, wide liquid range, high thermal stability, and good ionic conductivity. Due to these valuable properties, ionic liquids have generated attention due to their potential application as green and eco-friendly chemicals to replace traditional volatile organic solvents [4,5,6].

Hassan and co-workers investigated the antibacterial activity of pyridinium-, phosphonium-, and imidazolium-based ionic liquids. Most ionic liquids showed good antibacterial properties, but ILs that consist of the imidazolium cation were even more antibacterial than the positive control [7,8,9]. Phenylacetic acid is an organic compound containing a phenyl and carboxyl functional group used in penicillin G production. It was added to the culture of *Penicillium* species to increase the production of penicillin G. 4-methoxyphenylacetic acid is phenylacetic acid with a 4-methoxy substituent, and it is used as an intermediate for pharmaceutical synthesis [10]. Also, benzoic acid is commonly used as a food preservative [11]. This is due to the low price and a broad spectrum of antimicrobial activity [12]. The antibacterial effect is what connects the listed substances.

In this paper, ionic liquids formed by the combination of cation (1-butyl-3-methylimidazolium) and various anions that individually showed a significant antimicrobial effect (phenylacetate, benzoate, and 4-methoxyphenylacetate) were synthesized. The structures of 1-butyl-3-methylimidazolium phenylacetate, [Bmim][Phe], 1-butyl-3-methylimidazolium benzoate, [Bmim][Ben], and 1-butyl-3-methylimidazolium 4-methoxyphenylacetate, [Bmim][CH_3_OPhe] were confirmed by FTIR and ^1^H and ^13^C NMR spectra. Based on the measured density, viscosity, and conductivity, interactions between cations and anions were discussed. Using an in silico approach, the other physicochemical properties were calculated and compared with those obtained experimentally. Also, the antimicrobial effect of these ionic liquids was examined, discussed, and correlated with the ILs’ structures.

## 2. Results and Discussion

### 2.1. Experimental Density, Viscosity and Conductivity of Studied ILs

Densities (*d*), viscosities (*ƞ*), and electrical conductivities (*κ*) of [Bmim][Phe], [Bmim][Ben], and [Bmim][CH_3_OPhe] were measured in the temperature range from *T* = (293.15 to 323.15) K, at atmospheric pressure (*p* = 0.1 MPa). The results are shown in Table 1.

**Table 1:** Density (*d*), electrical conductivity (*κ*), molar conductivity ($\lambda_{m}$), and viscosity (*η*) of studied ILs in temperature range *T* = (293.15–323.15) K at atmospheric pressure, *p* = 0.1 MPa.

*T* (K)	*d* (g∙cm^−3^)	*κ* (mS∙cm^−1^)	$\lambda_{m}$ (S·cm^2^·mol^−1^)	*η* (mPa·s)
[Bmim][Phe]				
293.15	1.09608	0.036	0.009	705.14
298.15	1.09286	0.074	0.019	461.22
303.15	1.08959	0.139	0.035	308.93
308.15	1.08626	0.221	0.056	215.01
313.15	1.08285	0.317	0.080	155.76
318.15	1.07942	0.421	0.107	116.77
323.15	1.07608	0.538	0.137	92.61
[Bmim][Ben]				
293.15	1.10804	0.193	0.045	775.41
298.15	1.10493	0.303	0.071	508.13
303.15	1.10170	0.450	0.106	346.85
308.15	1.09841	0.650	0.154	237.55
313.15	1.09502	0.910	0.216	170.07
318.15	1.09155	1.578	0.376	124.73
323.15	1.08810	2.124	0.508	95.15
[Bmim][CH_3_OPhe]				
293.15	1.12334	0.068	0.018	1215.11
298.15	1.12008	0.119	0.032	766.18
303.15	1.11678	0.211	0.057	496.26
308.15	1.11340	0.323	0.088	336.43
313.15	1.10995	0.459	0.126	236.58
318.15	1.10636	0.619	0.170	172.06
323.15	1.10276	0.811	0.224	130.95

**Figure 1:**
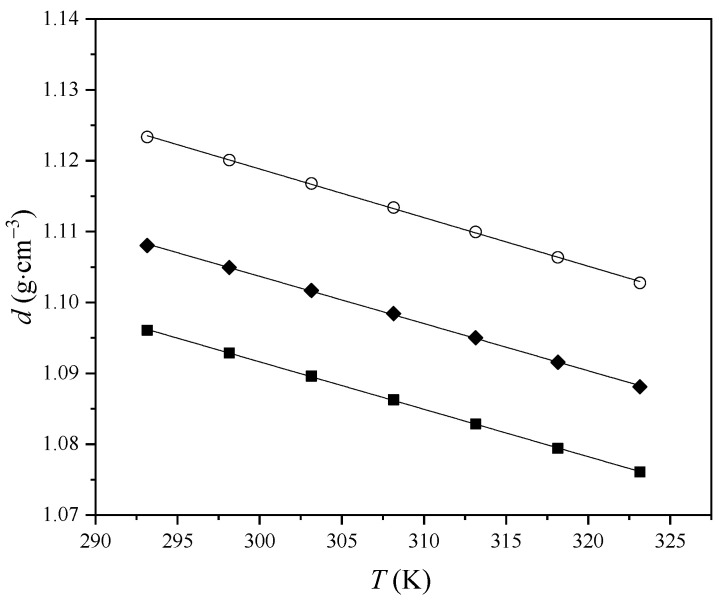
Variation in ILs’ density with temperature: ■, [Bmim][Phe]; ♦, [Bmim][Ben]; ○, [Bmim][CH_3_OPhe].

Variation in experimental density with the temperature is graphically presented in Figure 1.

As can be seen from Figure 1, the densities of pure ionic liquids linearly decrease with increasing temperature. The densities of pure ionic liquids follow the trend: [Bmim][Phe] < [Bmim][Ben] < [Bmim][CH_3_OPhe]. Density depends on cation–anion interactions and molecular packing. The results indicate that the introduction of a methoxy functional group in the benzene ring has a greater effect on the density growth than the shorter alkyl chain on the anion. It can be seen that ionic liquid density increases when the alkyl chain length of the anion decreases. This can be explained by the fact that as the chain length increases, the non-polar regions take up more space, leading to a lower overall IL density [12]. IL with methoxy group has the highest density, and this indicates that the [Bmim][CH_3_OPhe] has the best packing. It was found that a relatively long ether chain (e.g., more than three oxygen atoms) could reduce the IL density, but introducing only one ether group into a shorter alkyl chain can increase density [13].

From the measured values, the thermal expansion coefficient, *α_p_,* of pure ionic liquids is calculated using the equation [14]:

(1) $$\alpha_{p}=-\frac{1}{d}{\left(\frac{\partial d}{\partial T}\right)}_{p,m}$$

**Figure 2:**
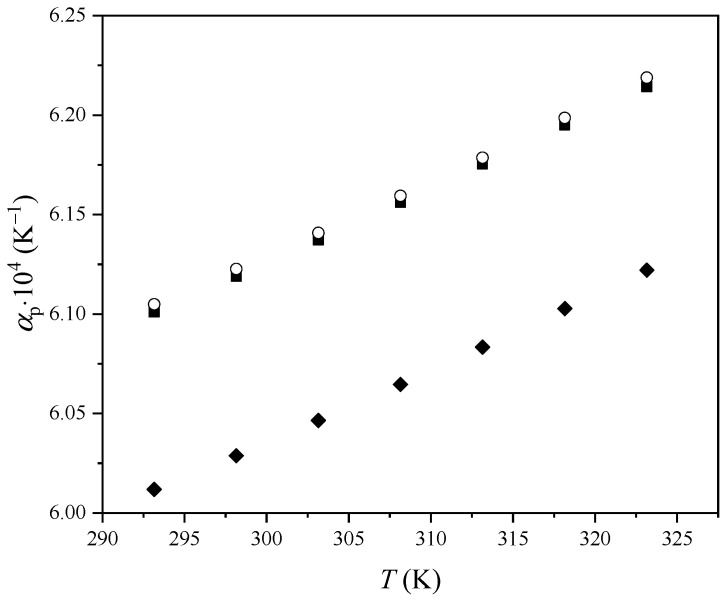
Variation in thermal expansion coefficients *α_p_* of ILs with temperature: ■, [Bmim][Phe]; ♦, [Bmim][Ben]; ○, [Bmim][CH_3_OPhe].

The obtained values of the *α_p_* are shown in Table 2, and their variation with the temperature is presented in Figure 2.

**Figure 3:**
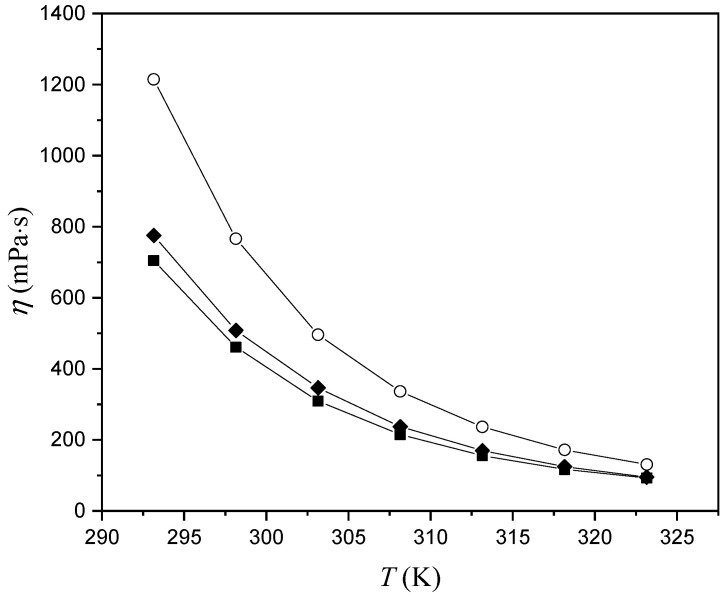
Variation in the ILs’ viscosity (*η*) with temperature: ■, [Bmim][Phe]; ♦, [Bmim][Ben]; ○, [Bmim][CH_3_OPhe].

As can be seen from Figure 2, *α_p_* increases with the increase in temperature. Values for *α_p_* show the trend [Bmim][Ben] < [Bmim][Phe] < [Bmim][CH_3_OPhe]. The thermal expansion coefficient indicates the change in the liquid volume as the temperature changes. From Figure 2 and Table 2, it can be observed that the *α_p_* values of [Bmim][CH_3_OPhe] and [Bmim][Phe] are similar, while the *α_p_* values of [Bmim][Ben] are significantly lower. The difference between the thermal expansion coefficient of the [Bmim][Ben] relative to [Bmim][Phe] and [Bmim][CH_3_OPhe] reveals that *α_p_* increases as the alkyl chain of the anion increases. A relatively long ether chain can reduce the thermal expansion coefficient, but a shorter ether chain has no such effect (Figure 2) [15]. Variation in viscosity with temperature is presented in Figure 3.

From Figure 3, it can be concluded that the viscosity decreases with increasing temperature. This happened because, at higher temperatures, the molecules became more active due to thermal motion, thus reducing the intermolecular force between them and resulting in decreased viscosity. Conversely, at lower temperatures, stronger intermolecular forces between the molecules led to greater internal friction and higher viscosity. The highest viscosity shows [Bmim][CH_3_OPhe], while the lowest is observed in the case of [Bmim][Phe]. Results show that the presence of the methoxy group on the benzene ring increases the viscosity of ionic liquids. Viscosity describes the interactions in the system. The ionic liquid will be more viscous if the interactions in the system are stronger and vice versa. With increasing temperature, interactions are weaker, inducing the reduction of viscosity and improvement of ion fluidity.

**Figure 4:**
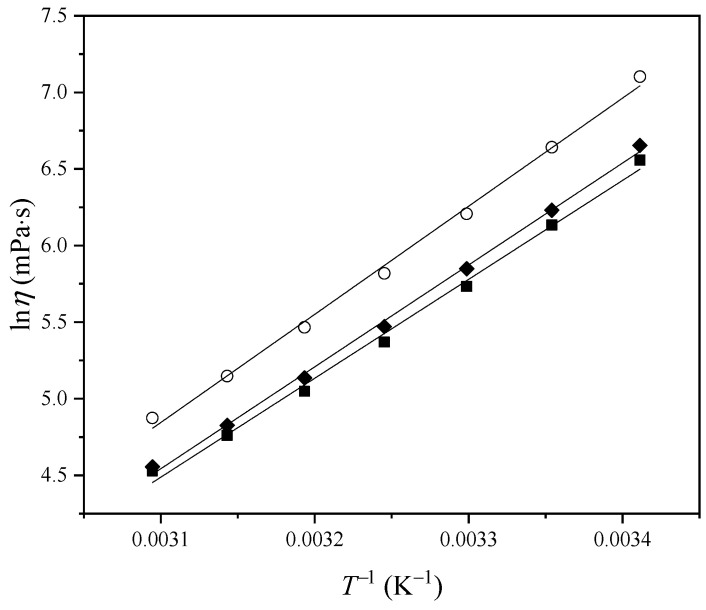
Variation in ln *η* with *T*^−1^: ■, [Bmim][Phe]; ♦, [Bmim][Ben]; ○, [Bmim][CH_3_OPhe].

Variation in viscosity with temperature was fitted using the logarithmic form of the Arrhenius equation (Figure 4) [16]:

(2) $$\ln\eta =-\frac{E_{a_{1}}}{RT}+\ln C$$

where *C* is the pre-exponential coefficient, *E*_a1_ is the activation energy of viscous flow, and *R* is the universal gas constant. The obtained activation energy values were 776.89 kJ·mol^−1^ for [Bmim][Phe], 800.25 kJ·mol^−1^ in the case of [Bmim][Ben], and 849.23 kJ·mol^−1^ for [Bmim][CH_3_OPhe]. It can be seen that the activation energy of viscous flow decreases with the addition of a methylene group on the anion but increases with the addition of one methoxy group.

**Figure 5:**
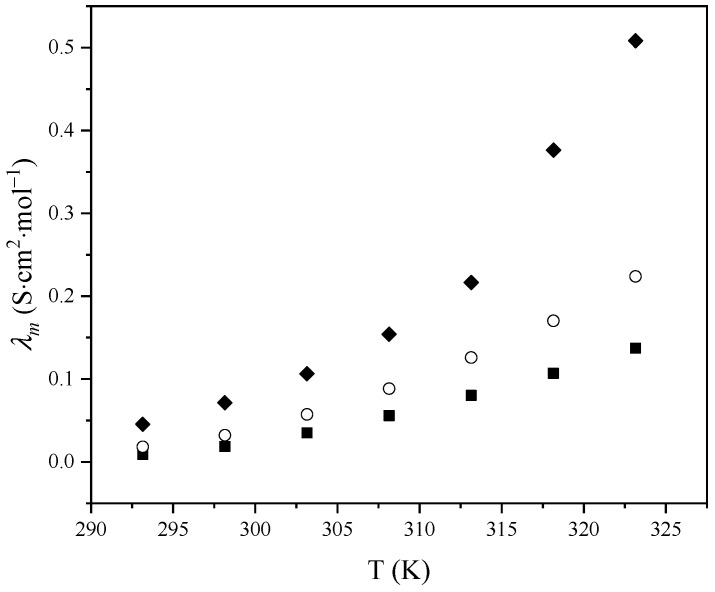
Variation in molar conductivity of pure ionic liquids with temperature: ■, [Bmim][Phe]; ♦, [Bmim][Ben]; ○, [Bmim][CH_3_OPhe].

**Figure 6:**
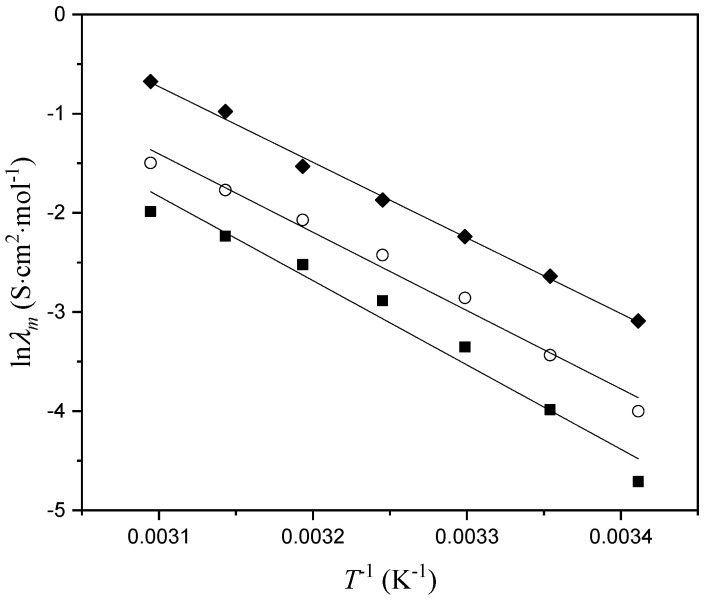
Variation in ln *λ_m_* viscosity with *T*^−1^: ■, [Bmim][Phe]; ♦, [Bmim][Ben]; ○, [Bmim][CH_3_OPhe].

Molar conductivity, $\lambda_{m}$ was calculated from the measured electrical conductivity using the equation:

(3) $$\lambda_{m}=\frac{\kappa }{c}$$

where $\lambda_{m}$ is molar conductivity, *κ* is electrical conductivity, and *c* is a molar concentration. Variation in molar conductivity with temperature is presented in Figure 5. The obtained dependence was fitted using the logarithmic form of the Arrhenius equation (Figure 6):

(4) $$\ln\lambda_{m}=-\frac{E_{a_{2}}}{RT}+\ln C$$

where *E_a2_* is the conductivity activation energy. The calculated values were 1024.85 kJ·mol^−1^ for [Bmim][Phe], 918.26 kJ·mol^−1^ for [Bmim][Ben], and 942.85 kJ·mol^−1^ in the case of [Bmim][CH_3_OPhe].

It can be observed that the molar conductivity increases with temperature. Ionic liquid with benzoate anion has the highest molar conductivity, followed by [Bmim][CH_3_OPhe], and finally, IL with phenylacetate anion has the lowest molar conductivity values.

Based on the Walden rule, Angell and co-workers have described a qualitative approach to the question: “How ionic is this ionic liquid?”. Angell has quantified such deviations by measuring the vertical distance to the KCl line and denoting these as ∆*W* [17,18].

(5) $$∆W=\log\eta^{-1}-\log\lambda_{m}$$

**Figure 7:**
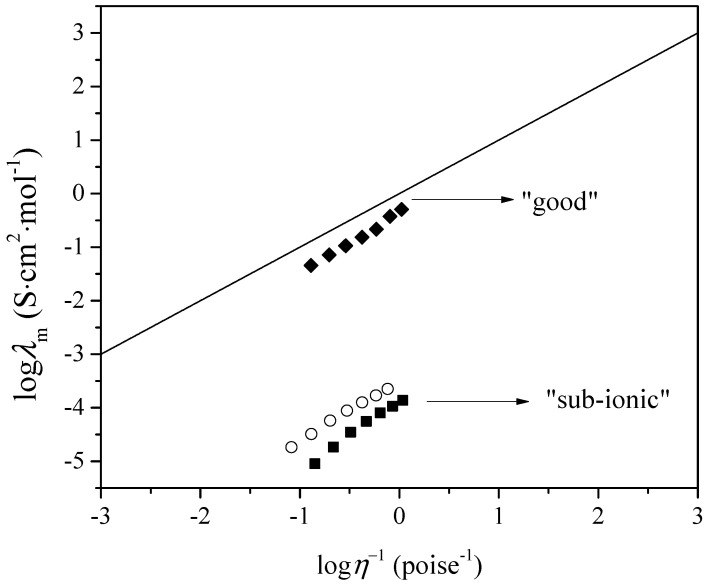
Walden plot in temperature range *T* = (293.15–323.15) K for: ■, [Bmim][Phe]; ♦, [Bmim][Ben]; ○, [Bmim][CH_3_OPhe]. The line represents values of 0.01 mol·dm^–3^ KCl aqueous solution.

To examine the ionicity of synthesized ionic liquids, the Walden plot was applied based on experimental values of viscosity and molar conductivity. The relation between molar conductivity and viscosity can be demonstrated by the equation:

(6) $$\log\lambda_{m}=\log C+\alpha \log\frac{1}{\eta }$$

(7) $$\alpha =100\cdot 10^{-∆W}$$

where *η*^−1^ is fluidity and *α* is the slope of the line in the Walden plot, which reflects the decoupling of the ions, *C*. The Walden plot is presented in Figure 7.

Figure 7 shows that all three ionic liquids fall below the ideal KCl line, indicating incomplete ionization. The vertical distance to the line (∆*W*) is a quantitative measure of ionicity. The Walden plot classifies ionic liquids into super-ionic, good, poor, and sub-ionic categories. Ionic liquids above the KCl line are classified as super-ionic ILs. The second group, close to the KCl line (0.1 < ∆*W* < 0.5), suggests the presence of almost mobile ions. The third group, lower on the plot, includes poor ionic liquids (0.5 < ∆*W* < 1.0) with specific ion interactions and pronounced H-bonds. Sub-ionic ILs fall far below the expected line for 100% ion dissociation (∆*W* > 1.0). Both [Bmim][Phe] and [Bmim][CH_3_OPhe] are far from the ideal KCl line in the group of sub-ionic ILs, indicating a lower degree of ionicity due to strong interactions in these ILs compared to [Bmim][Ben], which can be classified in the category of good ILs.

### 2.2. Chemometric Analysis of Similarities

Similarities and dissimilarities among the studied ILs were analyzed in the space of their experimentally determined physicochemical parameters: density (*d*), electrical conductivity (*κ*), molar conductivity ($\lambda_{m}$), viscosity (*η*), and thermal expansion coefficient (*α_p_*), together with other in silico-calculated physicochemical properties such as boiling point, melting point, critical temperature, critical pressure, critical volume, Gibbs free energy, lipophilicity descriptor, molar refractivity, total polar surface area, Crippen’s lipophilicity descriptor, Crippen’s molar refractivity, and solubility in water. The results of the HCA are presented in Figure S7.

Based on the presented double dendrograms in Figure S7, it can be found that the most similar ILs are [Bmim][Phe] and [Bmim][Ben] in the space of the variables *d*, *η*, and in silico physicochemical descriptors, since those ILs are placed in the same cluster, while [Bmim][CH_3_OPhe] significantly differs from them. On the other hand, [Bmim][Phe] and [Bmim][CH_3_OPhe] are similar in terms of *κ*, $\lambda_{m}$, and *α_p_* parameters. The double dendrograms presented in Figure S7 are based on raw or non-scaled data, considering the fact that all the data used are on the same scale; an exception is the clustering based on in silico physicochemical properties where the descriptors are on different scales, so the proportional scaling was applied. The following step included the HCA based on the scaled data using Z-scores. The obtained double dendrograms are presented in Figure S8. Here, the same clustering can be observed as in Figure S8—the ILs [Bmim][Phe] and [Bmim][Ben] have the most similar physicochemical features in terms of *d*, *η*, and in silico physicochemical descriptors, while [Bmim][Phe] and [Bmim][CH_3_OPhe] are similar considering *κ*, $\lambda_{m}$, and *α_p_* parameters. This is in agreement with previous discussion—introduction of the methoxy functional group in the benzene ring increases density and viscosity due to stronger cation–anion interactions and improves packing in the IL structure. The conclusions derived from the dendrograms concerning *κ* and $\lambda_{m}$ also confirmed the findings of the Walden plot (Figure 7) about better ionicity of [Bmim][Ben].

In both HCA approaches, the temperature-dependent physicochemical parameters (*d*, *η*, *κ*, $\lambda_{m}$, and *α_p_*) were used as variables whose values were measured in temperature intervals from (293.15 to 323.15) K. In double dendrograms, the vertical dendrogram shows the grouping of these parameters measured at different temperatures. Grouping the parameters measured at certain temperatures in the same cluster means that these values are similar. For example, the values of *κ* and $\lambda_{m}$ parameters measured at 318.15 K and 323.15 K are placed in the same cluster (Figure S7c,d), keeping in mind that [Bmim][Ben] has significantly higher *κ* and $\lambda_{m}$ parameters at those temperatures than other ILs. At temperatures between 293.15 K and 313.15 K, there is a parallel change in the parameters of all three ILs, but above 313.15 K, there is a significant discrepancy in the parameter values of [Bmim][Ben]. A similar phenomenon can be observed for *η* values of [Bmim][CH_3_OPhe] at temperatures 393.15 K and 398.15 K. These values are placed in the same vertical cluster in Figure S7b. This is in accordance with experimental results, since the temperature has a significant impact on cation–anion interactions, affecting the behavior and properties of ionic compounds. As temperature increases, the kinetic energy of the ions increases. This increased kinetic energy can overcome the electrostatic forces holding the cations and anions together, leading to a weakening of the interactions, and pronounced changes in physicochemical features can be observed.

### 2.3. Antimicrobial Activity of Ionic Liquids

**Figure 8:**
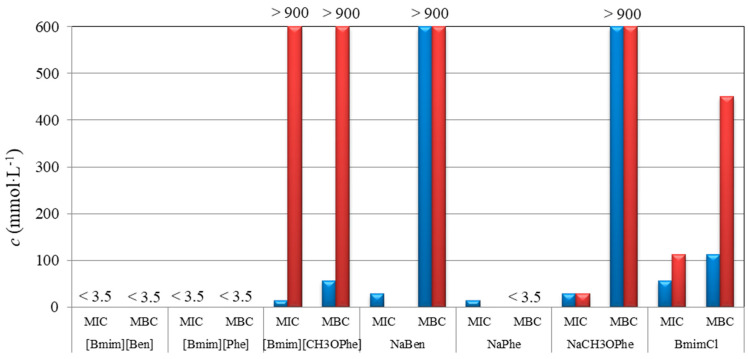
Antibacterial activity of tested ILs and corresponding sodium salts against *Escherichia coli* (blue), and *Pseudomonas aeruginosa* (red).

**Figure 9:**
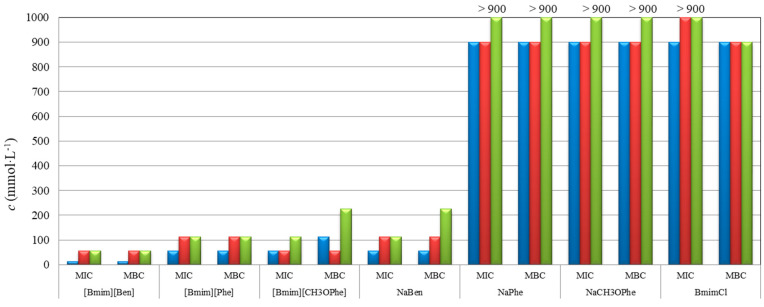
Antifungal activity of tested ILs and corresponding sodium salts against *Penicillium verrucosum* (blue), *Aspergillus flavus* (red), and *Aspergillus parasiticus* (green).

Results of minimum inhibitory concentration (MIC), minimum bactericidal (MBC), and minimum fungicidal (MFC) concentrations are presented in Table 3 and plotted in Figure 8 and Figure 9.

The most prominent observation was that studied ILs showed better antimicrobial activity than corresponding sodium salts containing the same anions. Among them, [Bmim][Ben] and [Bmim][CH_3_OPhe] have tenfold lower MIC, MBC, and MFC values than their corresponding sodium salts. Also, bacterial strains were more sensitive (MIC and MBC values varying from <3.5 mmol·L^−1^ to 56.3 mmol·L^−1^) to the tested compounds than filamentous fungi (MIC and MBC values from 14.1 mmol·L^−1^ to 900 mmol·L^−1^).

Within the tested bacterial strains, [Bmim][Ben], [Bmim][Phe], and NaPhe showed the best activity with MIC and MBC values for *E. coli* and *P. aeruginosa* lower than 3.5 mmol·L^−1^ (0.91 g·L^−1^, 0.96 g·L^−1^, 0.55 g·L^−1^, respectively), which was the lowest concentration tested. This is expected, since it is known that both phenylacetic and benzoic acids show good antibacterial activities. In the study of Hajfarajollah et al. [19], eleven 1-butil-3-methylimidazolium-based ILs were tested against *E. coli* and *P. aeruginosa,* and all except one showed MIC and MBC values in the range of 3.1–25.0 g·L^−1^ which indicates that the studied ILs possess better potential in antibacterial activity against those bacterial strains. Similar results were observed in the study of Anvari et al. [20], where 3-methylimidazolium-based ILs tested against *E. coli* and *P. aeruginosa* showed MIC and MBC values in the range of 0.39–25.0 g·L^−1^.

Figure 8 shows that [Bmim][Ben] and [Bmim][Phe] have greater antibacterial activities compared to [Bmim][CH_3_OPhe]. These results indicate that ionic liquid with methoxy group is a weaker antibacterial agent than [Bmim][Ben] and [Bmim][Phe] in the case of *E. coli*, and it has no effect on the *P. aeruginosa* in the tested concentration range. This means that adding a methoxy group reduces the antibacterial activity while adding a methylene group on the anion does not lead to a significant change.

Antifungal activity of studied ILs and corresponding sodium salts showed that [Bmim][Ben], [Bmim][Phe], [Bmim][CH_3_OPhe], and NaBen had the best activity (MIC and MFC values ranged from 14.1 to 225 mmol·L^−1^) among all the tested compounds and *Penicillium* strain was more sensitive (MIC and MFC from 14.1 to 113 mmol·L^−1^) than *Aspergillus* strains (MIC and MFC in the range of 56.3–225 mmol·L^−1^) as it can be seen from Figure 9. In the study of Karaman et al. [21], 1-butil-3-methylimidazolium chloride was tested against filamentous fungi (*Alternaria* sp.), and MIC and MFC values were found in the range of 53.0–106 mmol·L^−1^, showing that ILs containing halide anions do not express the remarkable influence on the ILs’ toxicity compared to those containing organic anions.

The antimicrobial activity is not significantly affected by elongating the shorter carbon chain (C1–C4), but extending the longer carbon chain (>C4) noticeably enhances the activity. Moreover, the addition of a benzene ring substantially reduces the minimal bactericidal concentration, as evidenced by our results and existing literature [22]. Also, by comparing the results obtained for the minimum concentration with the literature data [23], it is seen that replacing a small anion with a large, organic anion increases antimicrobial activity.

The highest antimicrobial activity and the lowest observed MIC, MBC, and MFC in the case of benzoate-based compound is expected since [Bmim][Ben] shows the highest lipophilicity (log*P*) and the lowest solubility (log*S*) among the studied ILs, which can be seen from the dendrograms in Figures S7f and S8f. Introduction of the methylene group between the benzene aromatic ring and carboxylate anion decreases lipophilicity of the [Phe]-anion, thus reducing antimicrobial activity. Decrease in the antimicrobial activity is observed in the case of [Bmim][CH_3_OPhe] compared to other two studied ILs since the introduction of the oxygen atoms in the cation structure significantly increases the hydrophilicity and solubility in water [24].

## 3. Materials and Methods

### 3.1. Synthesis

**Figure 10:**
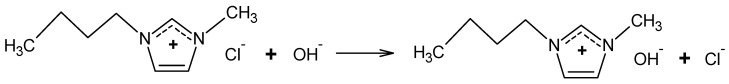
Schematic representation of [Bmim][OH] synthesis.

All reagents used for the synthesis of the studied ILs were used without purification. Their detailed specifications are given in Table S1 in the Supporting Materials of this manuscript. In the initial phase of synthesis, the 1-butyl-3-methylimidazolium chloride, [Bmim][Cl] was transformed to 1-butyl-3-methylimidazolium hydroxide, [Bmim][OH], using anion exchange raisin (Amberlite IRN78) (Figure 10). The procedure was repeated until the negative spot reaction for chloride. The obtained aqueous solution of [Bmim][OH] was used for further synthesis.

**Figure 11:**
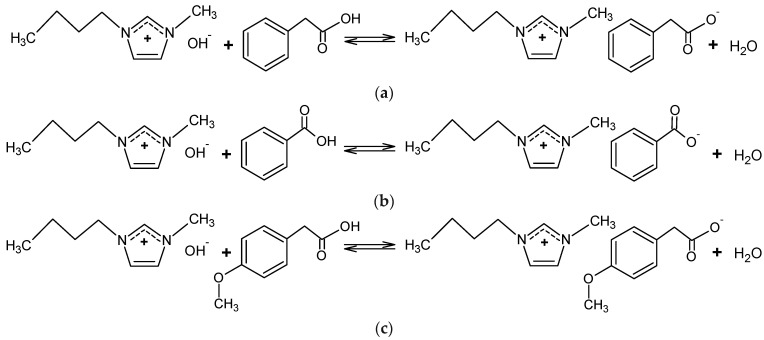
Schematic representation of: (**a**) [Bmim][Phe], (**b**) [Bmim][Ben], and (**c**) [Bmim][CH_3_OPhe] synthesis.

To obtain [Bmim][Phe], [Bmim][Ben], and [Bmim][CH_3_OPhe], potentiometric titrations using [Bmim][OH] were performed. The methanol solutions of phenylacetic acid, benzoic acid, and 4-methoxyphenylacetic acid were titrated with an aqueous solution of [Bmim][OH]. The obtained pH values at the equivalence point were 9.95 for [Bmim][Phe], 9.54 for [Bmim][Ben], and 11.81 in the case of [Bmim][CH_3_OPhe]. After titration water and methanol were removed by a rotary evaporator at 70 °C, the pale yellow-colored ionic liquids were obtained. The synthetic paths of the studied ILs are presented in Figure 11, and titration methods are used as described in the literature [25].

### 3.2. Structure Determination

The structure determination of the newly synthesized ionic liquids was performed by measuring their IR and NMR spectra. Results are presented in Figures S1–S6 in the Supplementary Materials with adequate assignation. NMR spectrum was recorded in D_2_O at 298 K on a Bruker Advance III 400 MHz spectrometer, Bruker Scientific Instruments, Billerica, MA, USA. Tetramethylsilane was used as an accepted internal standard for calibrating chemical shifts for ^1^H and ^13^C. ^1^H homodecoupling and the 2D COSY method were used routinely for the assignation of the obtained NMR spectra. Generally, the NMR spectra were recorded before and after density and viscosity measurements for all ILs. All ^13^C NMR spectra were assigned by the selective decoupling technique. The infrared spectrum was recorded from (4000 to 650) cm^−1^ on a Thermo-Nicolet Nexus 670 spectrometer fitted with a Universal ATR Sampling Accessory using ZnSe monocrystal, Thermo Fisher Scientific Inc., Waltham, MA, USA. The water content in the studied ILs was determined by the Karl Fischer titration method using the Metrohm 831 Karl Fischer coulometer, Metrohm, Riverview, FL, USA, and it was found to be less than 0.03% for all of the tested ionic liquids. This was taken into account for further calculations.

### 3.3. Physicochemical Properties: Experimental Density, Viscosity, and Electrical Conductivity

The density measurements of the synthesized IL were carried out at an atmospheric pressure of *p* = 0.1 MPa using the vibrating tube Rudolph Research Analytical DDM 2911 densimeter in the temperature range *T* = (293.15–323.15) K with an accuracy of ±0.00005 g·cm^−3^. The repeatability was within 0.01%, and the standard uncertainty was found to be less than 3 × 10^−4^ g·cm^−3^. Prior to each measurement, the instrument was calibrated at atmospheric pressure using triple distilled ultra-pure water and air at a temperature of 293.15 K. The densimeter has incorporated a Peltier thermostat, and the estimated relative standard uncertainty of temperature is less than 0.015 K. The viscosity-related errors in the density were automatically corrected. Each reported density value is the average of at least five measurements on the stated temperature. The total volume of the sample used for density measurements was approximately 1 cm^3^. The densimeter has already incorporated moisture adsorbent.

The viscosity of investigated ILs was measured using a Brookfield Viscometer DV II+ Pro thermostat within ±0.01 K and filled with about 15 cm^3^ of a pure ionic liquid. The spindle type (SC4-18) was immersed, and the rate per minute (RPM) was set to obtain a proper torque. The compartment made by the manufacturer is set to protect from moisture. A viscometer cell was calibrated using standard viscosity liquids (Supporting Materials Table S1) purchased from the manufacturer, which cover the viscosity range of all the investigated ILs. The viscosity of investigated ionic liquids was measured in the temperature range from *T* = (293.15 to 323.15) K with a rotation speed from 0.2 to 2 RPM. The mean of three measurements is presented as an experimental value with the relative standard uncertainty estimated to be about 0.02.

Electrical conductivity measurements of pure ILs were carried out in a Pyrex-cell with platinum electrodes in the temperature range *T* = (293.15–323.15) K on a conductivity meter Jenco 3107 using a DC signal. The experimental cell was calibrated with standard 0.1000 mol·dm^−3^ KCl solution and the cell constant equaled 1.0353 cm^−1^ (checked from time to time to control any possible evolution). Elimination of self-heating and ionization in the electrodes was achieved by performing at least ten measurements at 5 s intervals. The relative standard uncertainty for electrical conductivity was less than 1.5%. All obtained experimental values represent the mean value of three measurements.

### 3.4. In Silico Calculation of Physicochemical Properties of the Studied ILs

The other physicochemical properties of the analyzed ILs were calculated using an in silico approach and applying the ChemBioDraw Ultra v.13 program [26]. The calculations were based on a two-dimensional formula of the ILs whose structures were submitted to analysis as integrated cations and anions. The calculated molecular descriptors are the following: BP (boiling point, K), MP (melting point, K), CT (critical temperature, K), CP (critical pressure, bar), CV (critical volume, cm^3^·mol^−1^), GE (Gibbs free energy, kJ mol^−1^), logP (lipophilicity descriptor), MR (molar refractivity, cm^3^·mol^−1^), tPSA (total polar surface area, Å^2^), ClogP (Crippen’s lipophilicity descriptor), CMR (Crippen’s molar refractivity, cm^3^·mol^−1^), and logS (water solubility).

Hierarchical cluster analysis (HCA). The analysis of similarities and dissimilarities among the studied ILs in the space of considered variables was carried out by hierarchical cluster analysis with Ward’s minimum variance as the clustering method and Euclidean distances as the distance method. The results of the clustering were presented in the form of clustered heat maps or double dendrograms. The clustering was performed with and without scaling the data. The scaling method used was based on Z-scores.

### 3.5. Antimicrobial Activity of the Studied ILs

The synthesized ILs, together with the starting compounds, were tested against bacteria and filamentous fungi using an in vitro broth microdilution method according to adapted CLSI/EUCAST methodology (CLSI 2002, 2018; EUCAST 2008). Bacterial (*Escherichia coli* ATCC 8739, *Pseudomonas aeruginosa* ATCC 9027), and fungal (*Penicillium verrucosum* FCD0025, *Aspergillus flavus* FCB0046, *Aspergillus parasiticus* FCD0050) strains were obtained from the culture collection of Microbiology Laboratory, Faculty of Sciences University of Novi Sad. Bacterial suspension with turbidity equivalent to a 0.5 McFarland standard was prepared in sterile distilled water corresponding to 1–2 × 10^8^ CFU·mL^−1^, while for filamentous fungi spore suspension containing at least 10^6^ spores was made in saline solution. Nutrient broth (Tm Media, India) was inoculated with bacterial suspensions at a ratio of 1:100 (resulting in 1–2 × 10^6^ CFU of bacteria/mL), while malt extract broth (Torlak, Serbia) was used for inoculation with spore suspensions at a ratio of 1:100 (resulting in 1–4 × 10^6^ CFU of spores/mL). Experiments were performed in triplicates in a sterile polypropylene 96-well microtiter plate with a rounded bottom (Spektar, Čačak, Serbia), where 51 µL of inoculated nutrient or malt broth was added into each well with 50 µL of the tested compounds. The concentration of the tested ILs and corresponding anions was in the range of 3.5–900 mmol. Microtiter plates were incubated for 24 h at 37 °C for bacteria and 72 h at 26 °C for filamentous fungi, and minimal inhibitory concentration (MIC) values were determined visually as the lowest concentration of the tested compound where no visible growth of microorganisms was detected compared with the growth control. Minimal bactericidal (MBC) and minimal fungicidal concentrations (MFC) of the tested compounds were determined by transferring the content of wells where no visible growth was detected to Petri plates with Nutrient agar and Malt extract agar and incubated for 24 h (bacteria) at 37 °C and 72 h at 26 °C (filamentous fungi). The lowest concentration that reduces the viability of the original bacterial/fungal inoculums by 99.9% was found to be MBC and MFC values.

## 4. Conclusions

In this work, the ionic liquids with 1-butyl-3-methylimidazolium cation containing three carboxylate-based anions—benzoate, phenylacetate, and 4-methoxyphenylacetate—were synthesized and characterized by measuring their density, viscosity, and electrical conductivity at different temperatures. The increase in the densities of pure ionic liquids follows the trend [Bmim][Phe] < [Bmim][Ben] < [Bmim][CH_3_OPhe] due to a strengthening of the cation–anion interactions and better molecular packing. A similar trend is observed in the case of the thermal expansion coefficient, and it was found that *α_p_* increases as the alkyl chain of the anion increases. The highest viscosity shows [Bmim][CH_3_OPhe], while the lowest is observed in the case of [Bmim][Phe], showing that the presence of the methoxy group on the benzene ring increases the viscosity of ionic liquids. The measured electrical conductivity of the studied ILs was used to calculate molar conductivity and afterwards the ionicity using a Walden plot. It was observed that both [Bmim][Phe] and [Bmim][CH_3_OPhe] can be classified in the group of so-called sub-ionic ILs, due to a lower degree of ionicity and strong interactions in these ILs compared to [Bmim][Ben], which can be classified in the category of ILs with good ionicity. The analysis of similarities and dissimilarities among the studied ILs and their physicochemical properties applying the hierarchical cluster analysis confirms our conclusions derived from the experimental data. The research found that the investigated ILs had significantly stronger antimicrobial effects compared to the corresponding sodium salts containing the same anions as the ILs. Replacing an inorganic cation with an organic, bulky cation enhanced the antimicrobial activity. Similarly, replacing the Cl^−^ ion with a large, organic anion had an identical effect. The highest antimicrobial activity against two bacterial (*E. coli* and *P. aeruginosa*) and three fungi (*P. verrucosum*, *A. flavus*, and *A. parasiticus*) strains is observed in the case of [Bmim][Ben], which shows the highest lipophilicity and the lowest water solubility among the studied ILs.

## Figures and Tables

**Table 2:** Thermal expansion coefficients of [Bmim][Phe], [Bmim][Ben], and [Bmim][CH_3_OPhe].

*T* (K)	*α_p_*·10^4^ (K^−1^)		
	[Bmim][Phe]	[Bmim][Ben]	[Bmim][CH_3_OPhe]
293.15	6.10	6.01	6.10
298.15	6.12	6.03	6.12
303.15	6.14	6.05	6.14
308.15	6.16	6.06	6.16
313.15	6.18	6.08	6.18
318.15	6.20	6.10	6.20
323.15	6.21	6.12	6.22

**Table 3:** MIC and MBC/MFC values (mmol·L^−1^) of the tested ILs and individual anions against bacteria and filamentous fungi.

Bacteria		[Bmim][Ben]	[Bmim][Phe]	[Bmim][CH_3_OPhe]	NaBen	NaPhe	NaCH_3_OPhe	BmimCl
*E. coli*	MIC	<3.5	<3.5	14.1	28.1	14.1	28.1	56.3
	MBC	<3.5	<3.5	56.3	>900	<3.5	>900	113
*P. aeruginosa*	MIC	<3.5	<3.5	>900	<3.5	<3.5	28.1	113
	MBC	<3.5	<3.5	>900	>900	<5	>900	450
Fungi								
*P. verrucosum*	MIC	14.1	56.3	56.3	56.3	900	900	900
	MFC	14.1	56.3	113	56.3	900	900	900
*A. flavus*	MIC	56.3	113	56.3	113	900	900	>900
	MFC	56.3	113	56.3	113	900	900	900
*A. parasiticus*	MIC	56.3	113	113	113	>900	>900	>900
	MFC	56.3	113	225	225	>900	>900	900

## Data Availability

Data are contained within the article and Supplementary Materials.

## Supplementary Materials

The following supporting information can be downloaded at: https://www.mdpi.com/article/10.3390/molecules29153668/s1, Table S1: Provenance and purity of the samples, Figure S1: ^1^H NMR and ^13^C NMR spectra of [Bmim][Phe], Figure S2: ^1^H NMR and ^13^C NMR spectra of [Bmim][Ben], Figure S3: ^1^H NMR and ^13^C NMR spectra of [Bmim][CH3OPhe], Figure S4: FTIR spectra of [Bmim][Phe], Figure S5: FTIR spectra of [Bmim][Ben], Figure S6: FTIR spectra of [Bmim][CH3OPhe]. Figure S7: The results of HCA of ILs are based on (a) density, (b) viscosity, (c) electrical conductivity, (d) molar conductivity, (e) thermal expansion coefficient, and (f) in silico physicochemical properties. Figure S8: The results of HCA of ILs based on scaled data using Z-scores of (a) density, (b) viscosity, (c) electrical conductivity, (d) molar conductivity, (e) thermal expansion coefficient, and (f) in silico physicochemical properties.
