# Supplementary material for: Experimental and In Silico Comparative Study of Physicochemical Properties and Antimicrobial Activity of Carboxylate Ionic Liquids
Source: Molecules. 2024 Aug 2;29(15):3668. doi: 10.3390/molecules29153668 (PMC11314197; doi:10.3390/molecules29153668)
Supplement: Supplementary file 1 [file molecules-29-03668-s001.zip › molecules-3128972-supplementary.pdf]

Supporting Materials

Experimental and In Silico Comparative Study of Physicochemical Properties and Antimicrobial Activity of Carboxylate Ionic Liquids

Nikolett Cakó Bagány[1], Eleonora Čapelja[1], Strahinja Kovačević[2], Maja Karaman[1], Sanja Podunavac-Kuzmanović[2], Slobodan Gadžurić[1*] and Sanja Belić[1]

[1]*University of Novi Sad, Faculty of Science, Trg D. Obradovića 3, 21000 Novi Sad, Serbia*

[2]*University of Novi Sad, Faculty of Technology, Bulevar Cara Lazara 1, 21000 Novi Sad, Serbia*

Table S1. Provenance and purity of the samples

Chemical name	Source	Mass fraction purity	Purification method
1-butyl-3-methylimidazolium chloride	Sigma Aldrich	98%**	None
Phenylacetic acid	Sigma Aldrich	99%**	None
Benzoic acid	Sigma Aldrich	≥ 99.5%**	None
4-methoxyphenylacetic acid	Sigma Aldrich	99%**	None
Methanol	J.T. Baker	> 99%**	None
1-butyl-3-methyimidazolium phenylacetate	Synthesis	≥ 98%*	Rotary evaporation followed by vacuum
1-butyl-3-methylimidazolium benzoate	Synthesis	≥ 98%*	Rotary evaporation followed by vacuum
1-butyl-3-methylimidazolium 4-methoxyphenylacetate	Synthesis	≥ 98%*	Rotary evaporation followed by vacuum

*Determined by NMR measurements.
**Provided by the supplier.

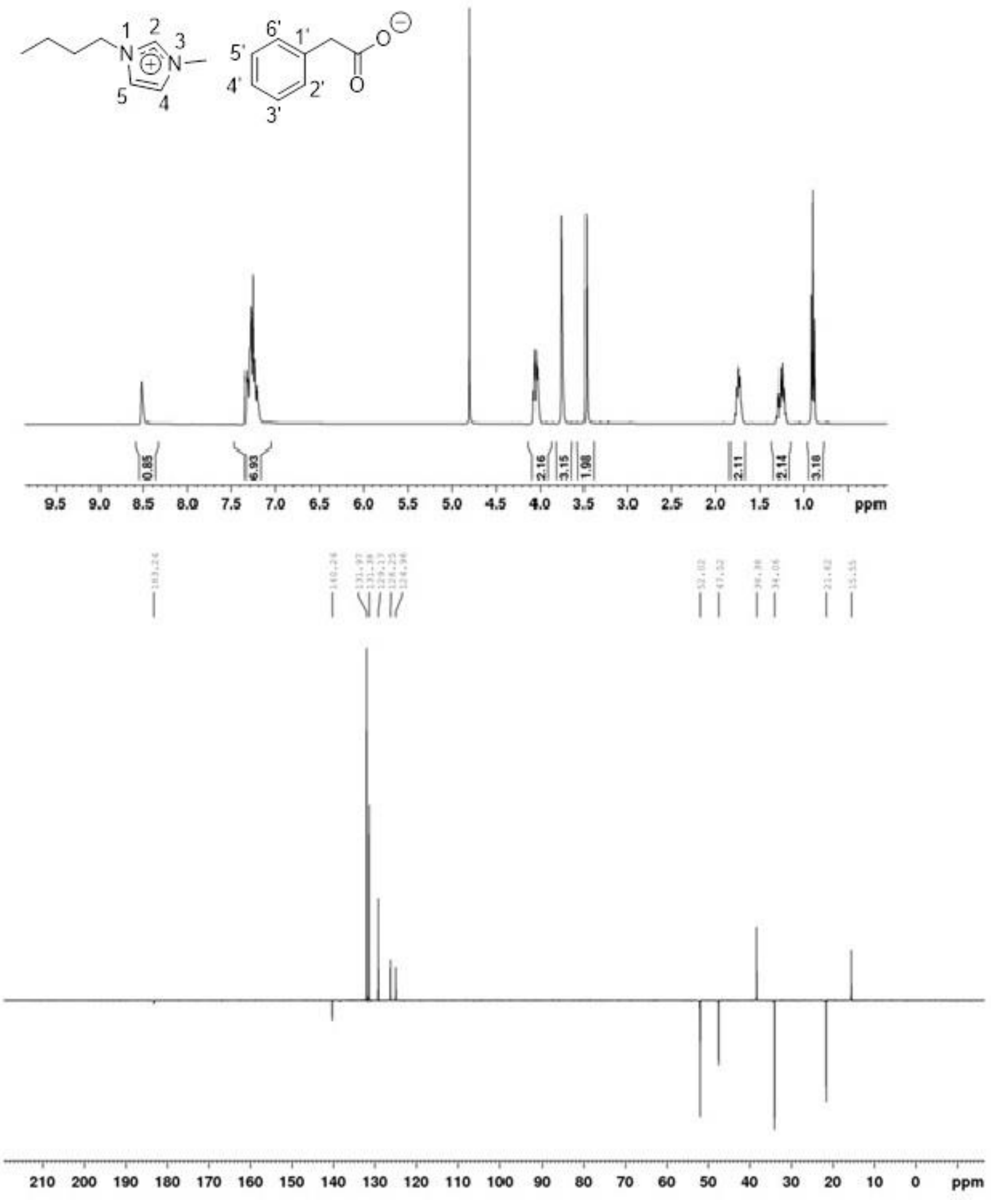

Figure S1. ^{1}H NMR and ^{13}C NMR spectra of [Bmim][Phe]

^{1}H NMR spectrum (D_2O): 8.52 (s, 1H, H-2); 7.16-7.37 (m, 7H, H-3′, H-5′, H-2′, H-6′, H-4′, H-4, H-5); 4.05 (t, 2H, NCH_2CH$_2$CH$_2$CH$_3$); 3.75 (s, 3H, NCH_3); 3.47 (s, 2H, CH_2CO$_2^-$); 1.75 (m, 2H, NCH$_2$CH_2CH$_2$CH$_3$); 1.26 (m, 2H, NCH$_2$CH$_2$CH_2CH$_3$); 0.89 (t, 3H, J = 7.1 Hz, NCH$_2$CH$_2$CH$_2$CH_3).
^{13}C NMR spectrum (D_2O): 183.24 (C=O); 140.24 (C-1′); 138.40 (t, $J_{2,D}$ = 33.6 Hz, C-2); 131.97 (C-2′and C-6′); 131.38 (C-3′ and C-5′); 129.17 (C-4′); 126.25 (C-4); 124.96 (C-5); 52.02 (NCH$_2$CH$_2$CH$_2$CH$_3$); 47.52 (CH$_2$CO$_2^-$); 38.38 (NCH$_3$); 34.06 (NCH$_2$$CH_2CH_2CH_3$); 21.62 (NCH$_2CH_2$$CH_2CH_3$); 15.55 (NCH$_2CH_2CH_2$$CH_3$).

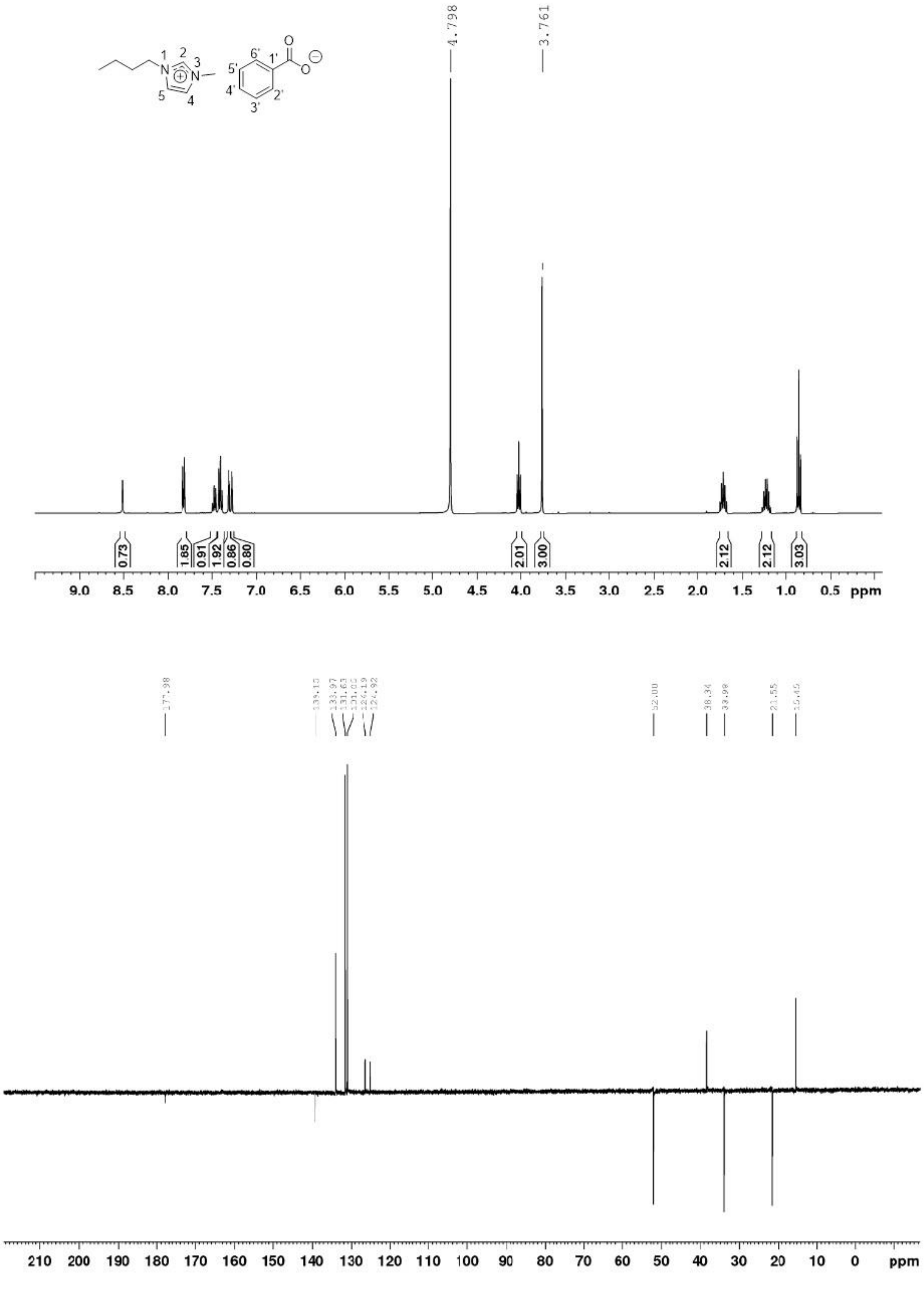

Figure S2. ^{1}H NMR and ^{13}C NMR spectra of [Bmim][Ben]

^{1}**H NMR** spectrum (D_2O): 8.51, (s, 1H, H-2); 7.37-7.85 (m, 5H, Ph-H); 7.16 (t, 1H, J = 1.8 Hz, H-5); 7.14 (t, 1H, J = 1.8 Hz, H-4); 4.02 (t, 2H, J = 7.3 Hz, $NCH_2CH_2CH_2CH_3$); 3.76 (s, 3H, NCH_3); 1.71 (m, 2H, $NCH_2CH_2CH_2CH_3$); 1.22 (m, 2H, $NCH_2CH_2CH_2CH_3$); 0.86 (t, 3H, J = 7.6 Hz, $NCH_2CH_2CH_2CH_3$).
^{13}**C NMR** spectrum (D_2O): 177.98 (C=O); 139.15 (C-1'); 138.36 (t, $J_{2,D}$ = 33.6 Hz, C-2); 133.97 (C-4'); 131.63 (C-2' and C-6'); 131.05 (C-3' and C-5'); 126.19 (C-4); 124.92 (C-5); 52.00 ($NCH_2CH_2CH_2CH_3$); 38.34 (NCH_3)**;** 33.98 ($NCH_2CH_2CH_2CH_3$); 21.55 ($NCH_2CH_2CH_2CH_3$); 15.45 ($NCH_2CH_2CH_2CH_3$).

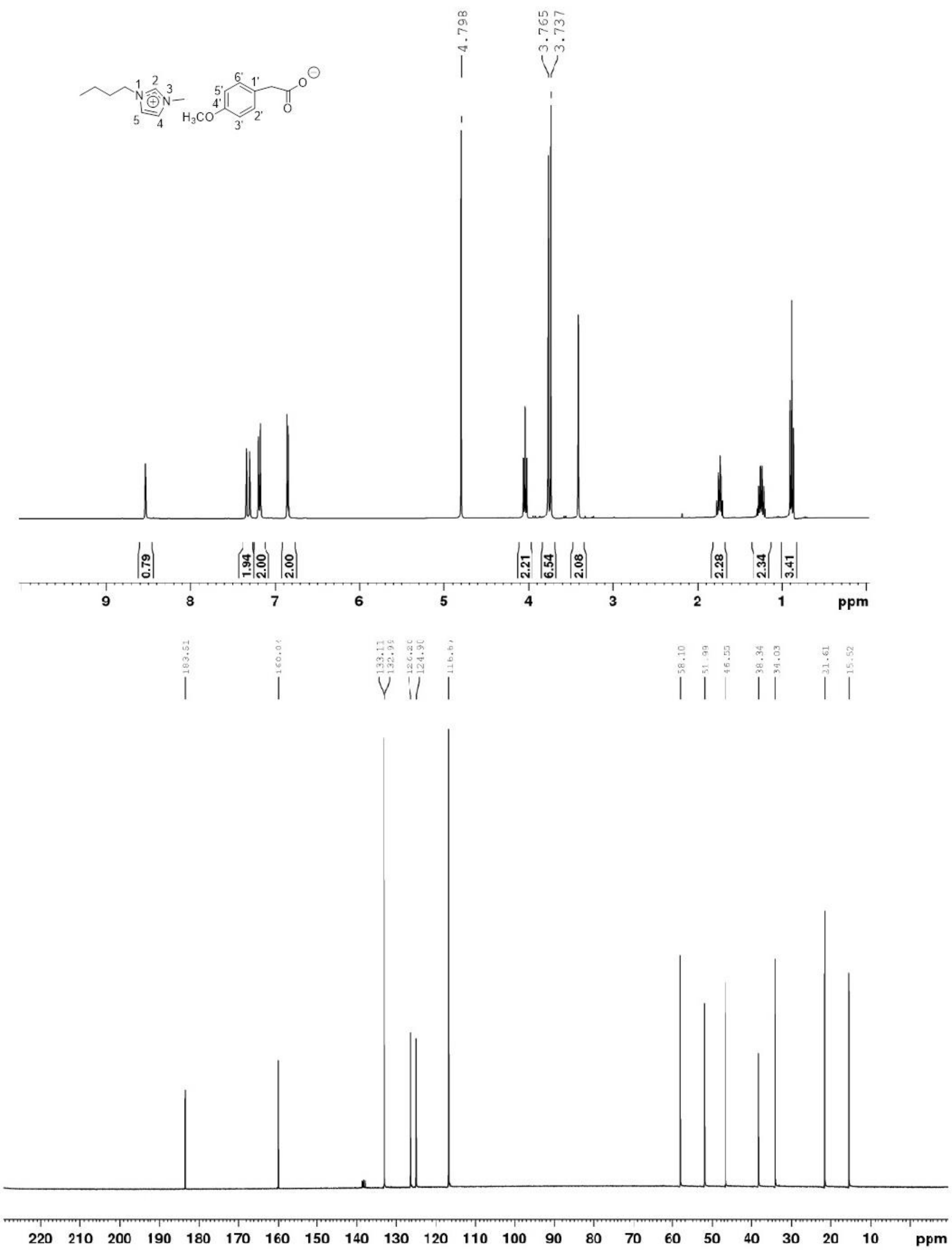

Figure S3. ^{1}H NMR and ^{13}C NMR spectra of [Bmim][CH_3OPhe]

^{1}**H NMR** spectrum (D_2O): 8.53 (s, 1H, H-2); 7.34 (t, 1H, J = 1.8 Hz, H-5); 7.30 (t, 1H, J= 1.8 Hz, H-4); 7.18 (d, 2H, J = 8.6 Hz, H-2′ and H-6′); 6.85 (d, 2H, J = 8.6 Hz, H-3′ and H-5′); 3.85 (t, 2H, J = 7.1 Hz, $NC\mathit{H}_2CH_2CH_2CH_3$); 3.76 (s, 3H, $NC\mathit{H}_3$); 3.74 (s, 3H, $OC\mathit{H}_3$); 3.41 (s, 2H, $C\mathit{H}_2CO_2^-$); 1.74 (m, 2H, $NCH_2C\mathit{H}_2CH_2CH_3$); 1.25 (m, 2H, $NCH_2CH_2C\mathit{H}_2CH_3$); 0.88 (t, 3H, J = 7.1 Hz, $NCH_2CH_2CH_2CH_3$).
^{13}**C NMR** spectrum (D_2O): 183.51 (C=O); 160.04 (C-4′); 138.19 (t, $J_{2,D}$ = 33.6 Hz, C-2); 133.11 (C-2′ and C-6′); 132.99 (C-1′); 126.20 (C-4); 124.90 (C-5); 116.67 (C-3′ and C-5′); 58.10 ($O\mathit{C}H_3$); 51.99 ($N\mathit{C}H_2CH_2CH_2CH_3$); 46.55 ($\mathit{C}H_2CO_2^-$); 38.34 ($N\mathit{C}H_3$); 34.03 ($NCH_2\mathit{C}H_2CH_2CH_3$); 21.61 ($NCH_2CH_2\mathit{C}H_2CH_3$); 15.52 ($NCH_2CH_2CH_2\mathit{C}H_3$).

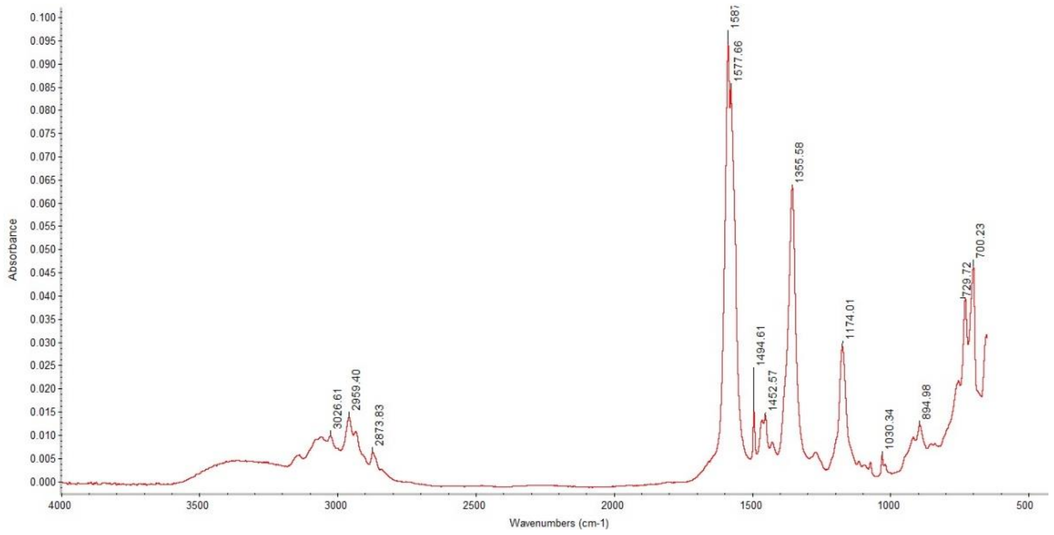

Figure S4. FTIR spectra of [Bmim][Phe]

3026 (sym. stretching vibration of N-CH_3); 2959 (sym. stretching vibration –CH_3 group bound to N); 1577 (sym. stretching vibration of the acetate ion, COO^- group of phenylacetate); 1355 (rocking vibration H-C-H of butyl group); 1174 (bending vibration of imidazolium ring); 700 (in-plane bending vibration of monosubstituted aromatic ring).

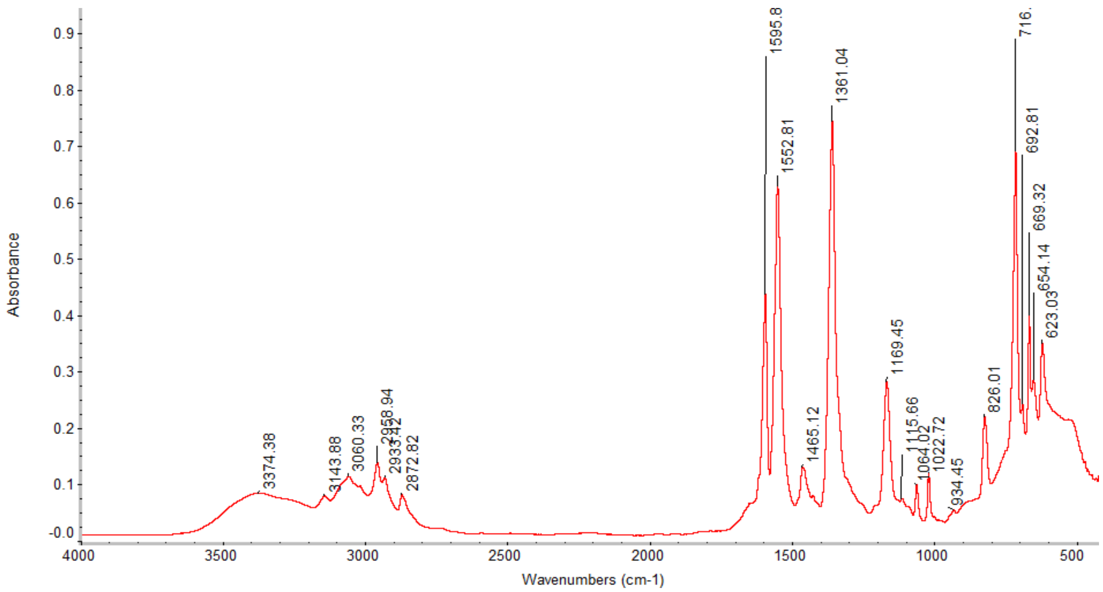

Figure S5. FTIR spectra of [Bmim][Ben]

2958 (sym. stretching vibration –CH_3 group bound to N); 1552 (asym. vibration of substituted benzoate); 1361 (rocking vibration H-C-H of butyl group); 1169 (bending vibration of imidazolium ring); 716 (in-plane bending vibration of monosubstituted aromatic ring).

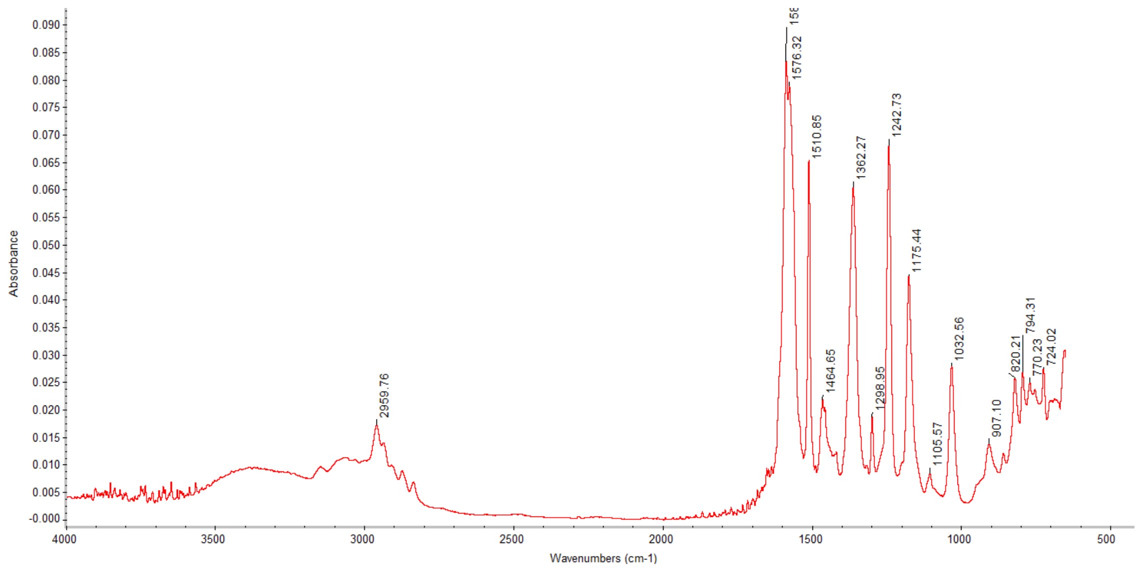

Figure S6. FTIR spectra of [Bmim][CH_3OPhe]

IR (neat): 2959 (sym. stretching vibration –CH_3 group bound to N), 1580 (sym. stretching vibration of the acetate ion, COO^- group of phenylacetate); 1362 (rocking vibration H-C-H of butyl group); 1242 (stretching vibration of aromatic ethers, aryl-O)； 1175 (bending vibration of imidazolium ring); 1105 (stretching vibration of alkyl-substituted ether, C-O)；770 (vibration of para disubstituted aromatic ring).

S7a)

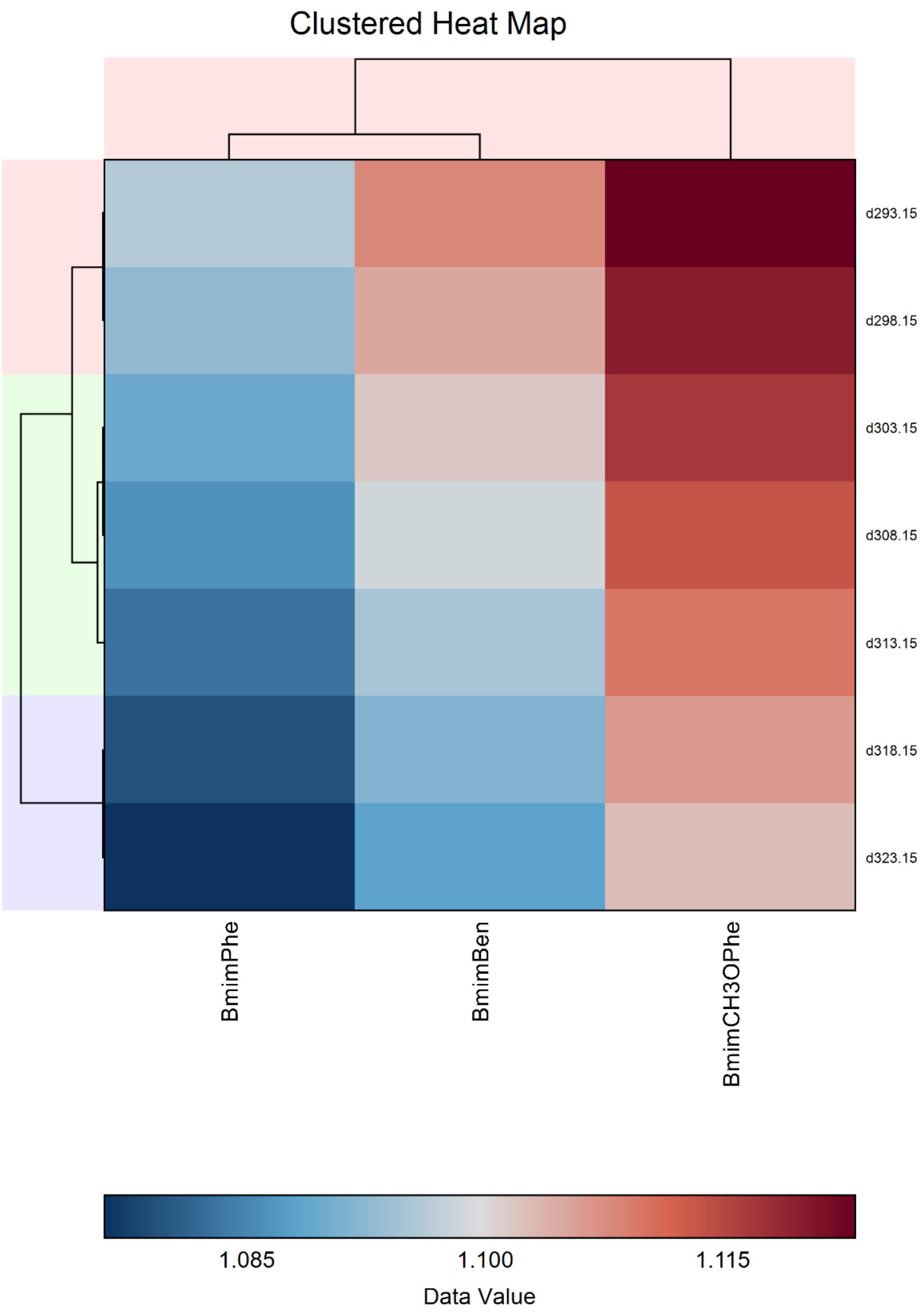
Clustered Heat Map
d293.15
d298.15
d303.15
d308.15
d313.15
d318.15
d323.15
BmimPhe
BmimBen
BmimCH3OPhe
1.085
1.100
1.115
Data Value

S7b)

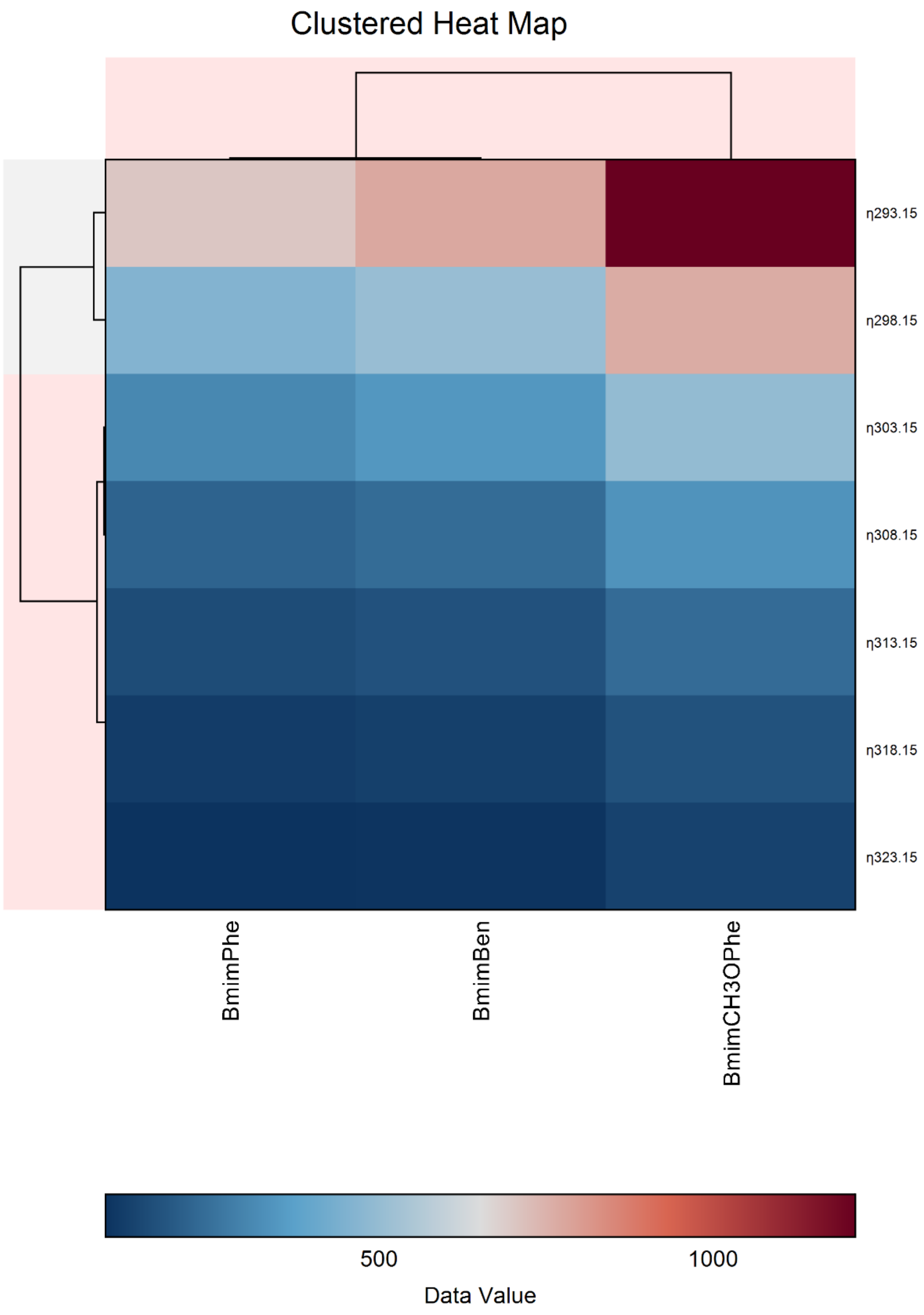
Clustered Heat Map
η293.15
η298.15
η303.15
η308.15
η313.15
η318.15
η323.15
BmimPhe
BmimBen
BmimCH3OPhe
500
1000
Data Value

S7c)

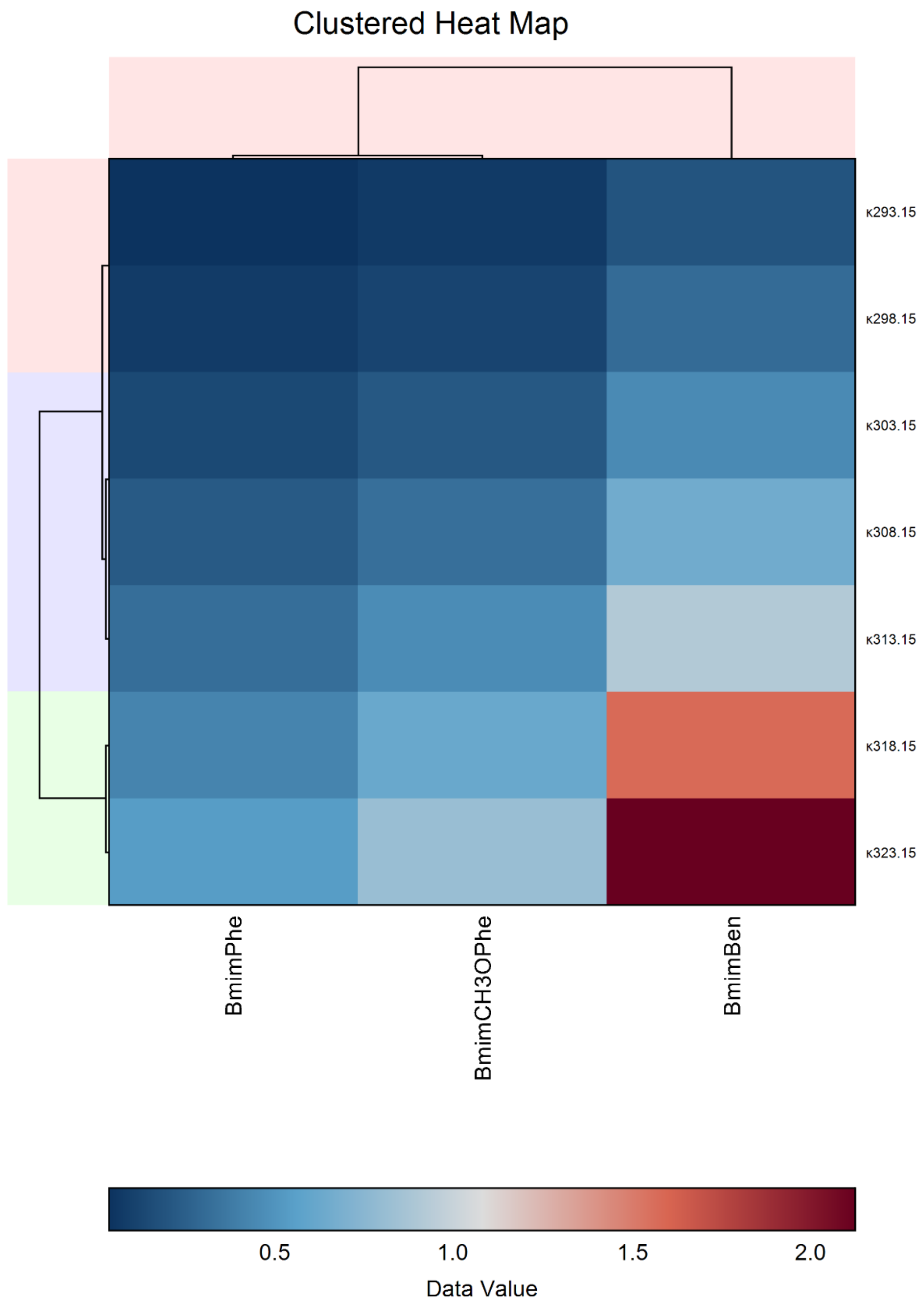
Clustered Heat Map
κ293.15
κ298.15
κ303.15
κ308.15
κ313.15
κ318.15
κ323.15
BmimPhe
BmimCH3OPhe
BmimBen
0.5
1.0
1.5
2.0
Data Value

S7d)

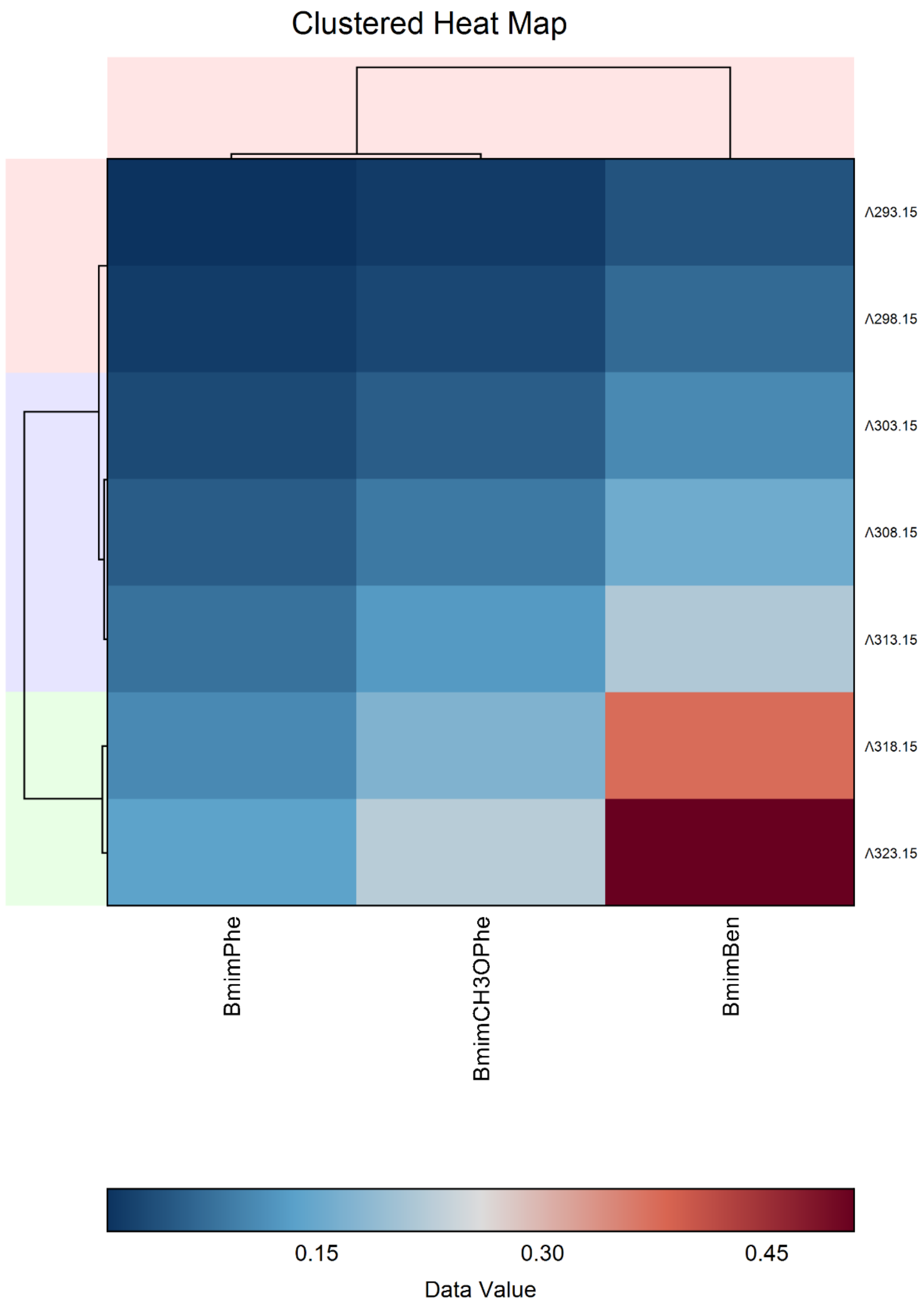
Clustered Heat Map
Λ293.15
Λ298.15
Λ303.15
Λ308.15
Λ313.15
Λ318.15
Λ323.15
BmimPhe
BmimCH3OPhe
BmimBen
0.15
0.30
0.45
Data Value

S7e)

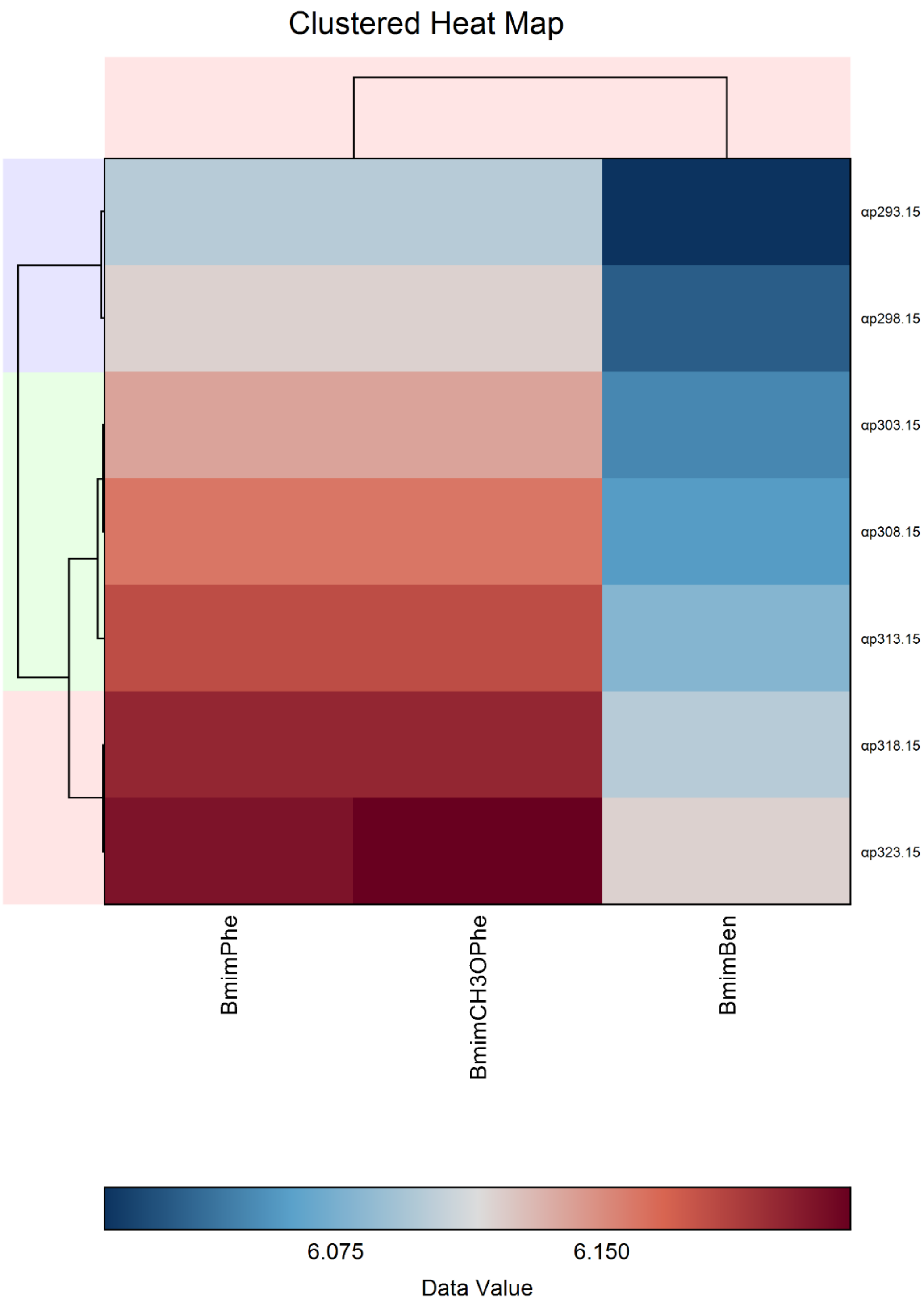
Clustered Heat Map
αp293.15
αp298.15
αp303.15
αp308.15
αp313.15
αp318.15
αp323.15
BmimPhe
BmimCH3OPhe
BmimBen
6.075
6.150
Data Value

S7f)

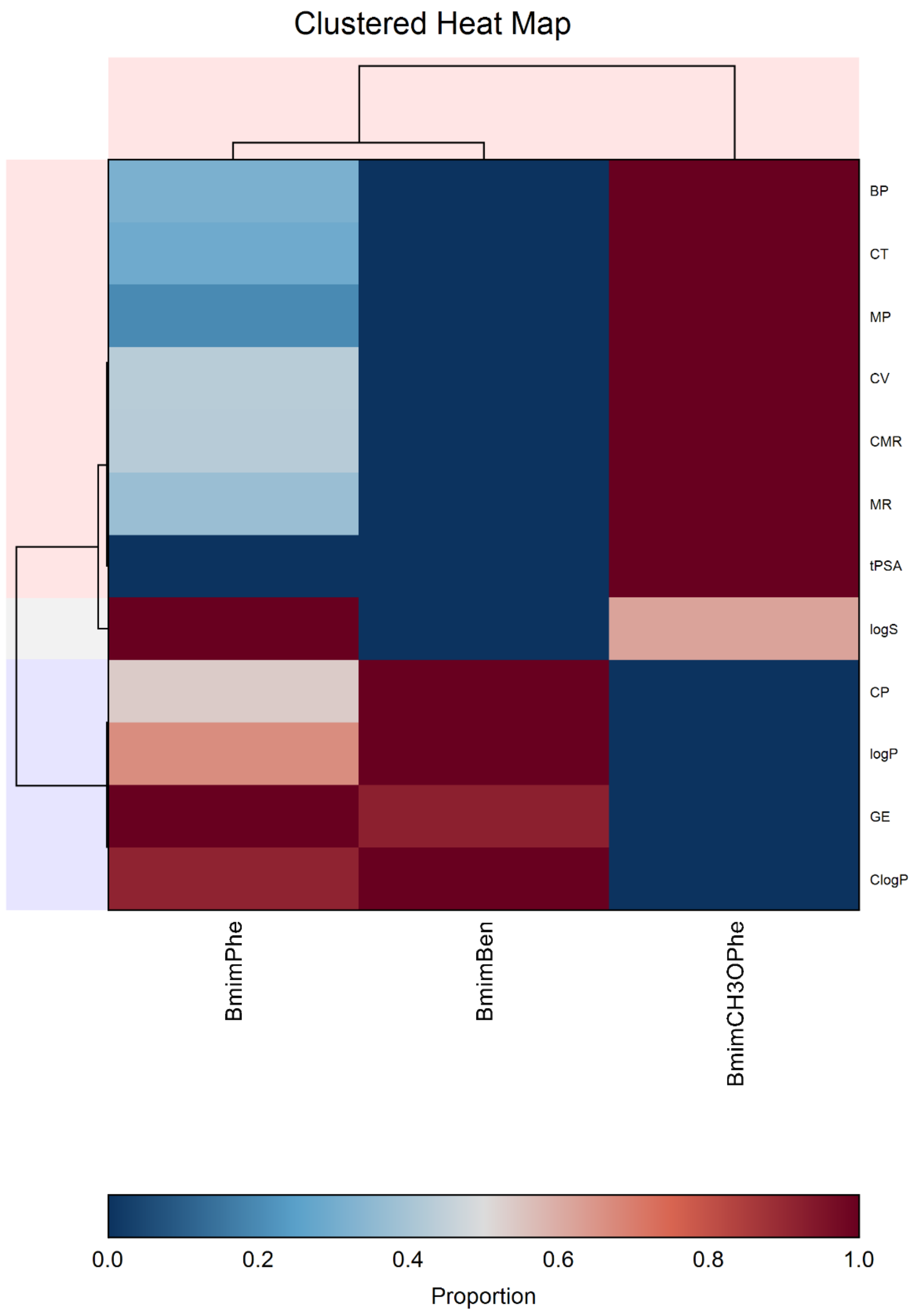

Figure S7. The results of HCA of ILs are based on a) density, b) viscosity, c) electrical conductivity, d) molar conductivity, e) thermal expansion coefficient, and f) in silico physicochemical properties.

S8a)

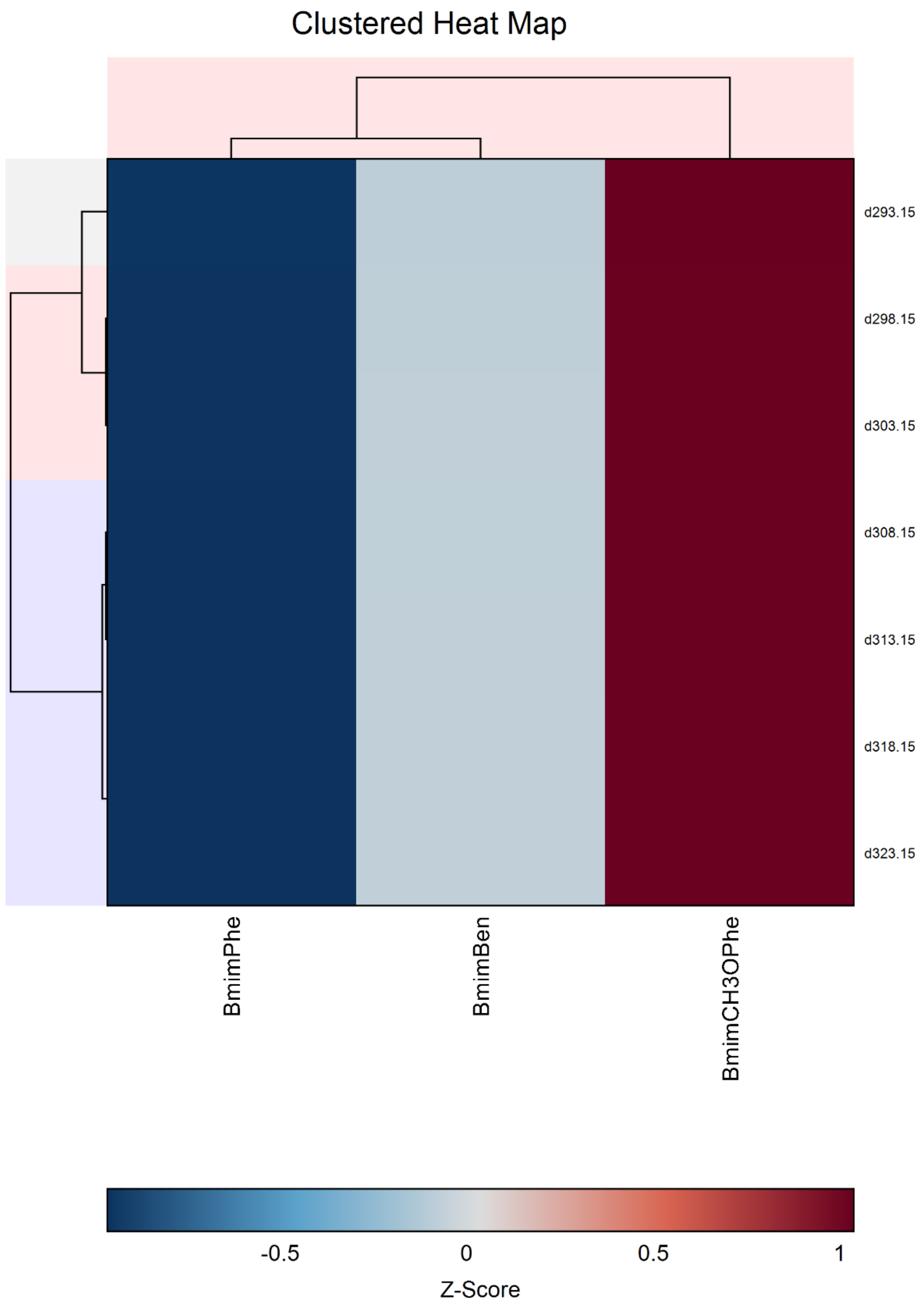
Clustered Heat Map
d293.15
d298.15
d303.15
d308.15
d313.15
d318.15
d323.15
BmimPhe
BmimBen
BmimCH3OPhe
-0.5
0
0.5
1
Z-Score

S8b)

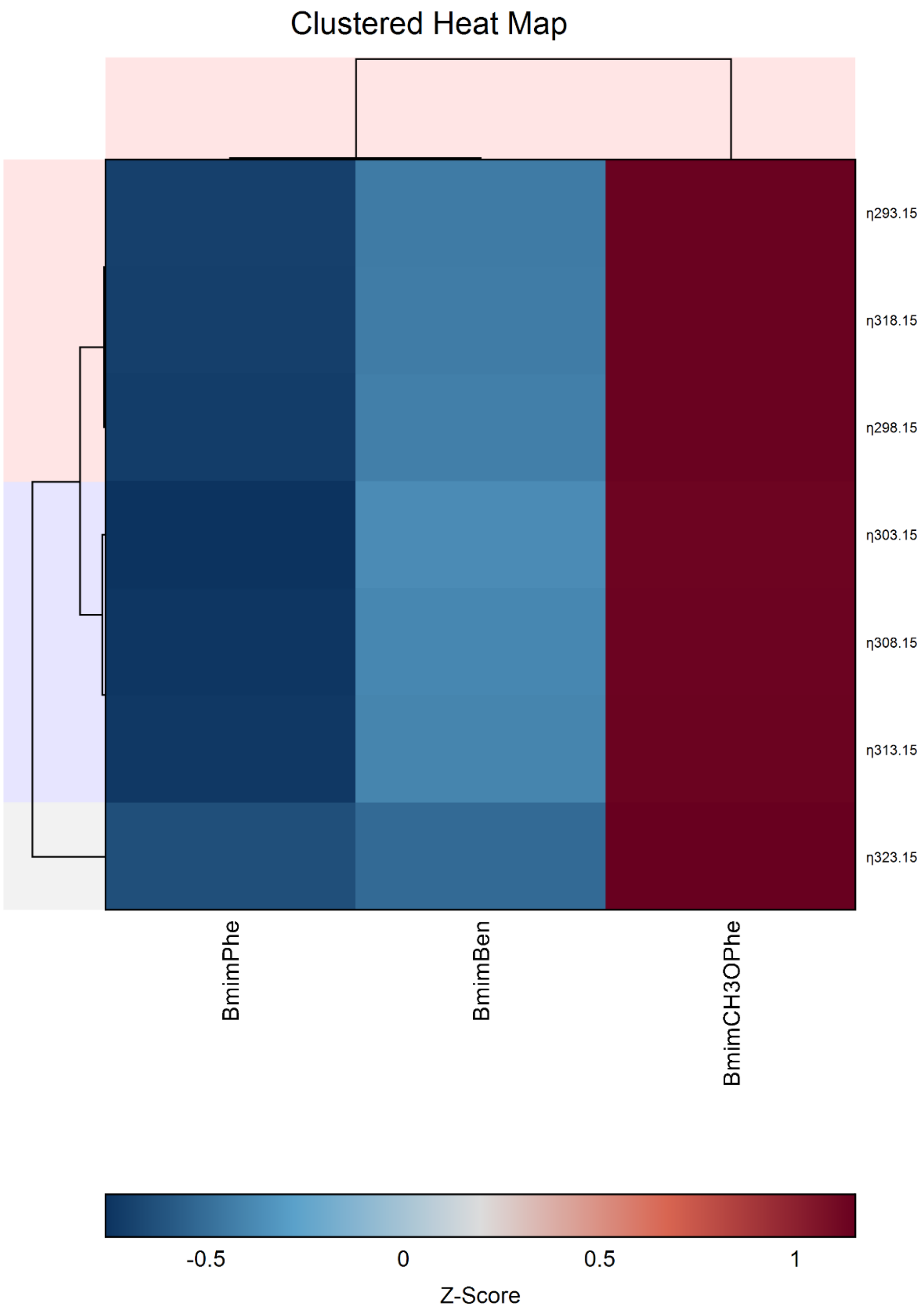
Clustered Heat Map
η293.15
η318.15
η298.15
η303.15
η308.15
η313.15
η323.15
BmimPhe
BmimBen
BmimCH3OPhe
-0.5
0
0.5
1
Z-Score

S8c)

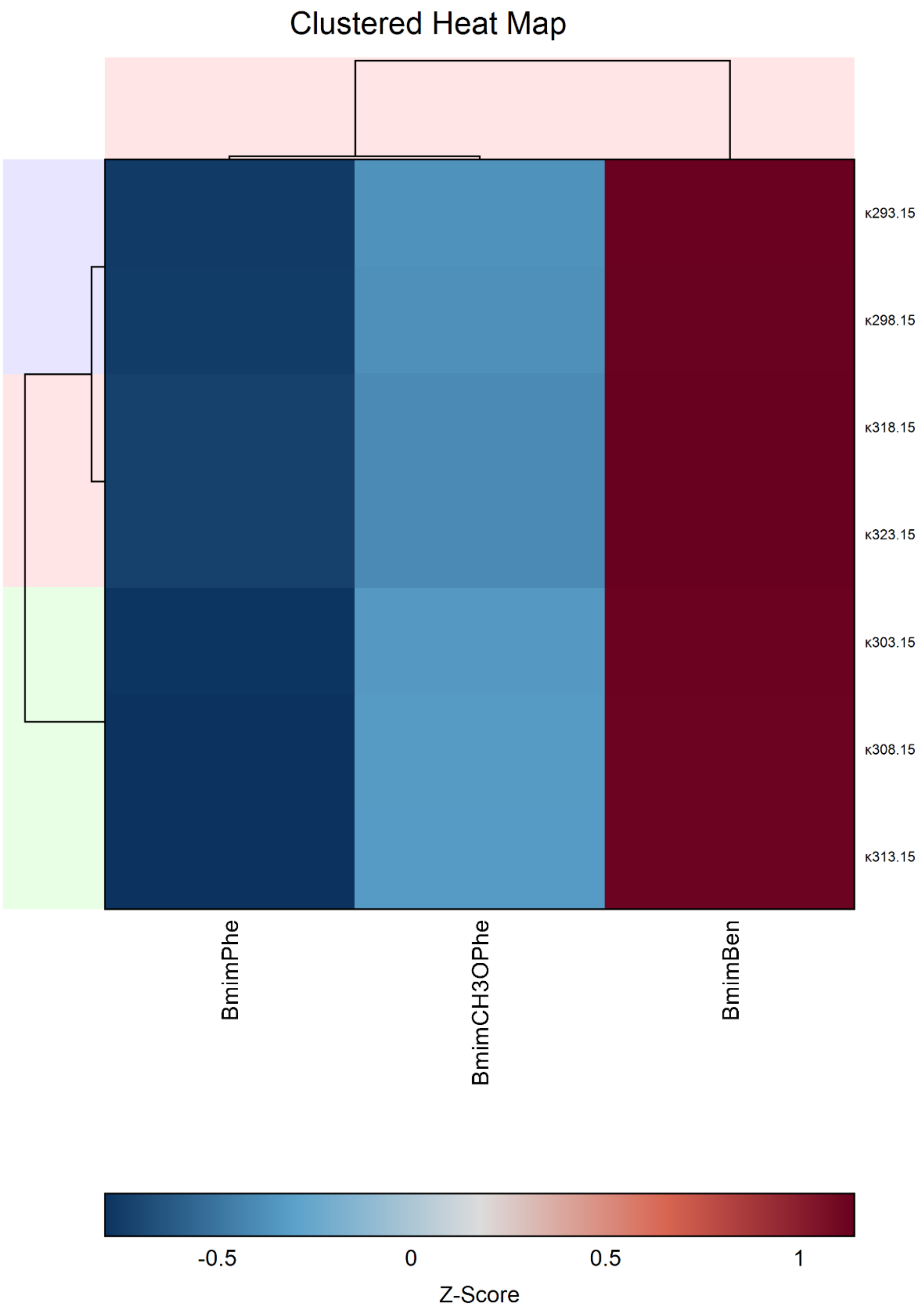
Clustered Heat Map
κ293.15
κ298.15
κ318.15
κ323.15
κ303.15
κ308.15
κ313.15
BmimPhe
BmimCH3OPhe
BmimBen
-0.5
0
0.5
1
Z-Score

S8d)

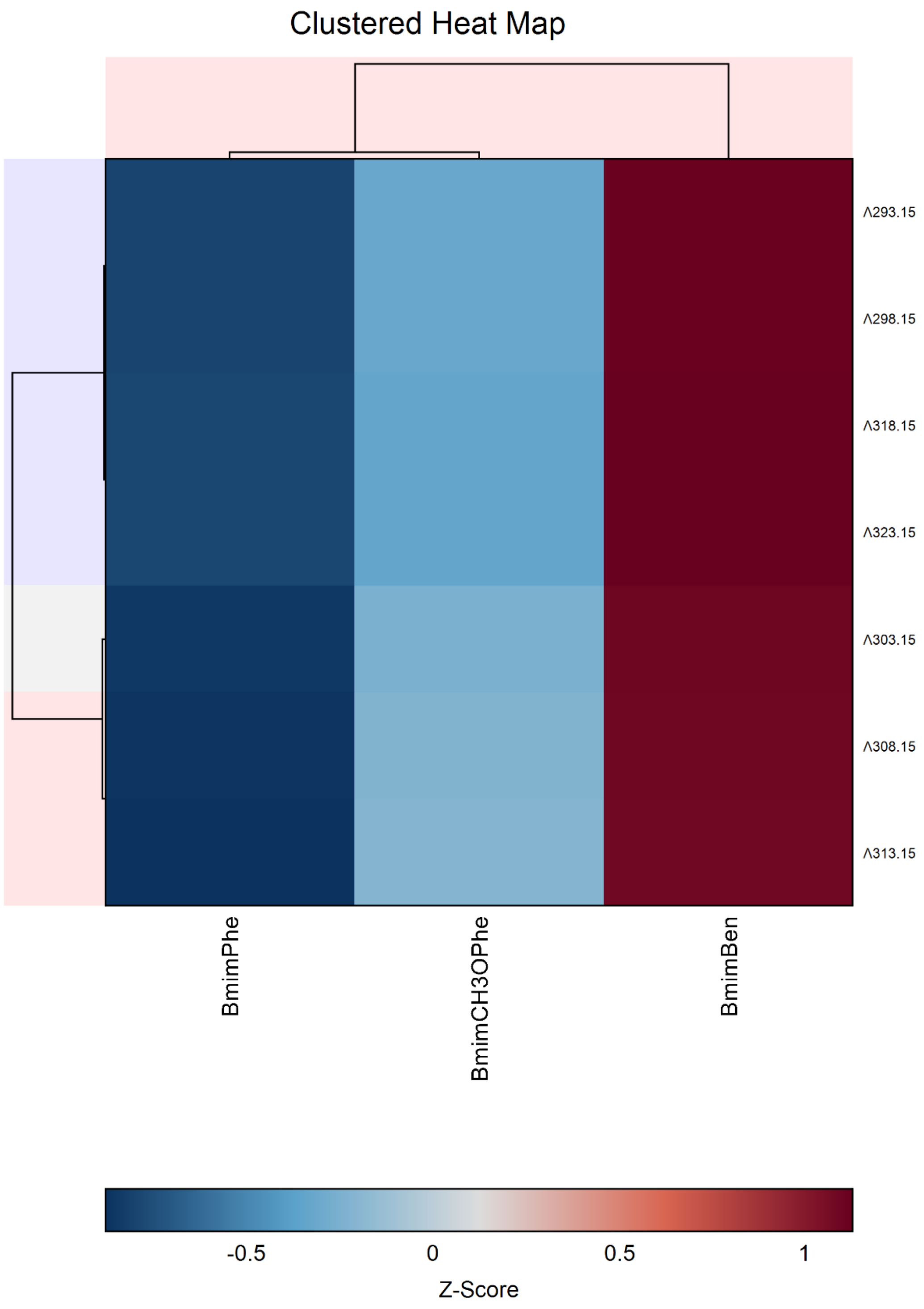

Clustered Heat Map
Λ293.15
Λ298.15
Λ318.15
Λ323.15
Λ303.15
Λ308.15
Λ313.15
BmimPhe
BmimCH3OPhe
BmimBen
-0.5
0
0.5
1
Z-Score

S8e)

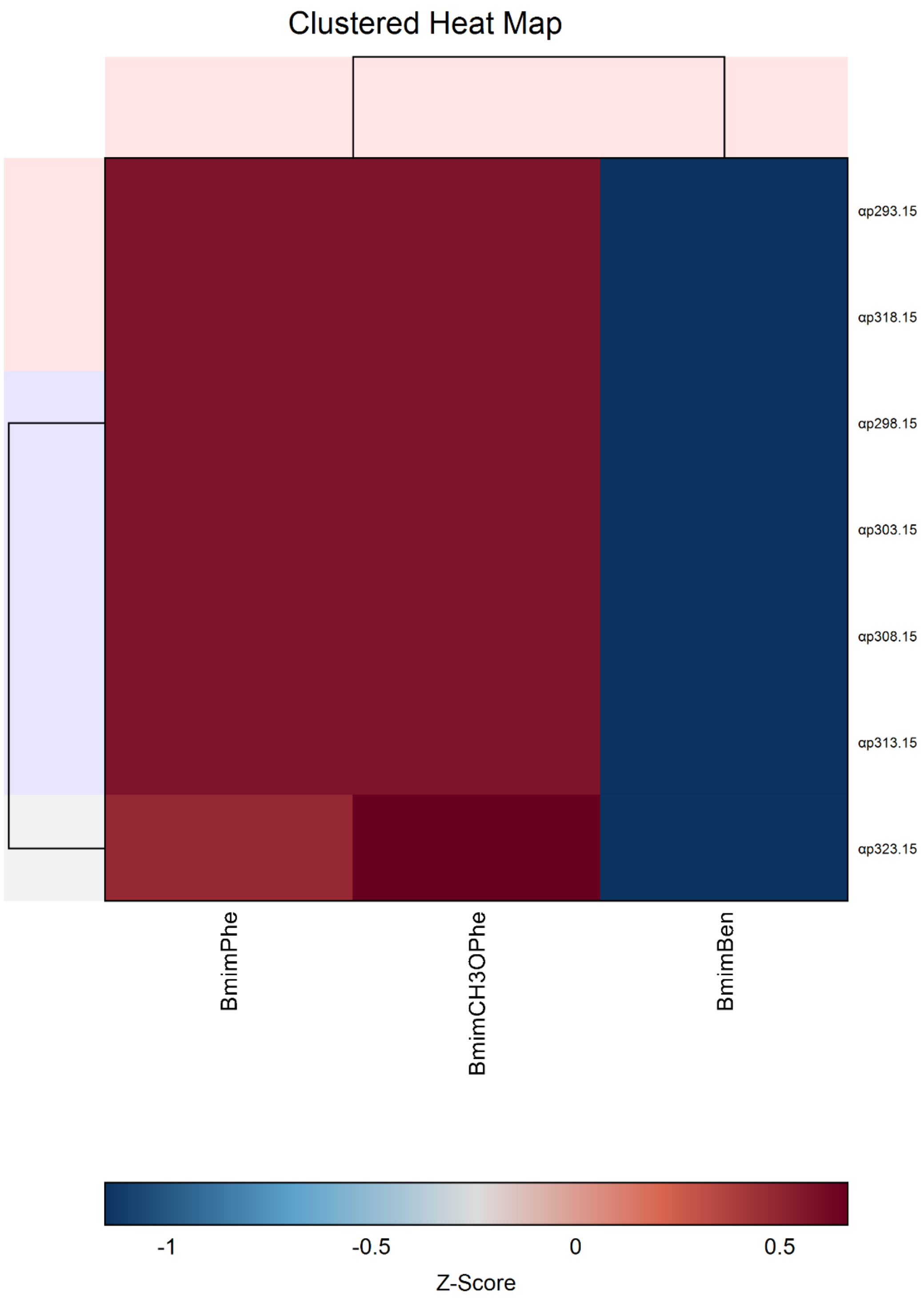
Clustered Heat Map
αp293.15
αp318.15
αp298.15
αp303.15
αp308.15
αp313.15
αp323.15
BmimPhe
BmimCH3OPhe
BmimBen
-1
-0.5
0
0.5
Z-Score

S8f)

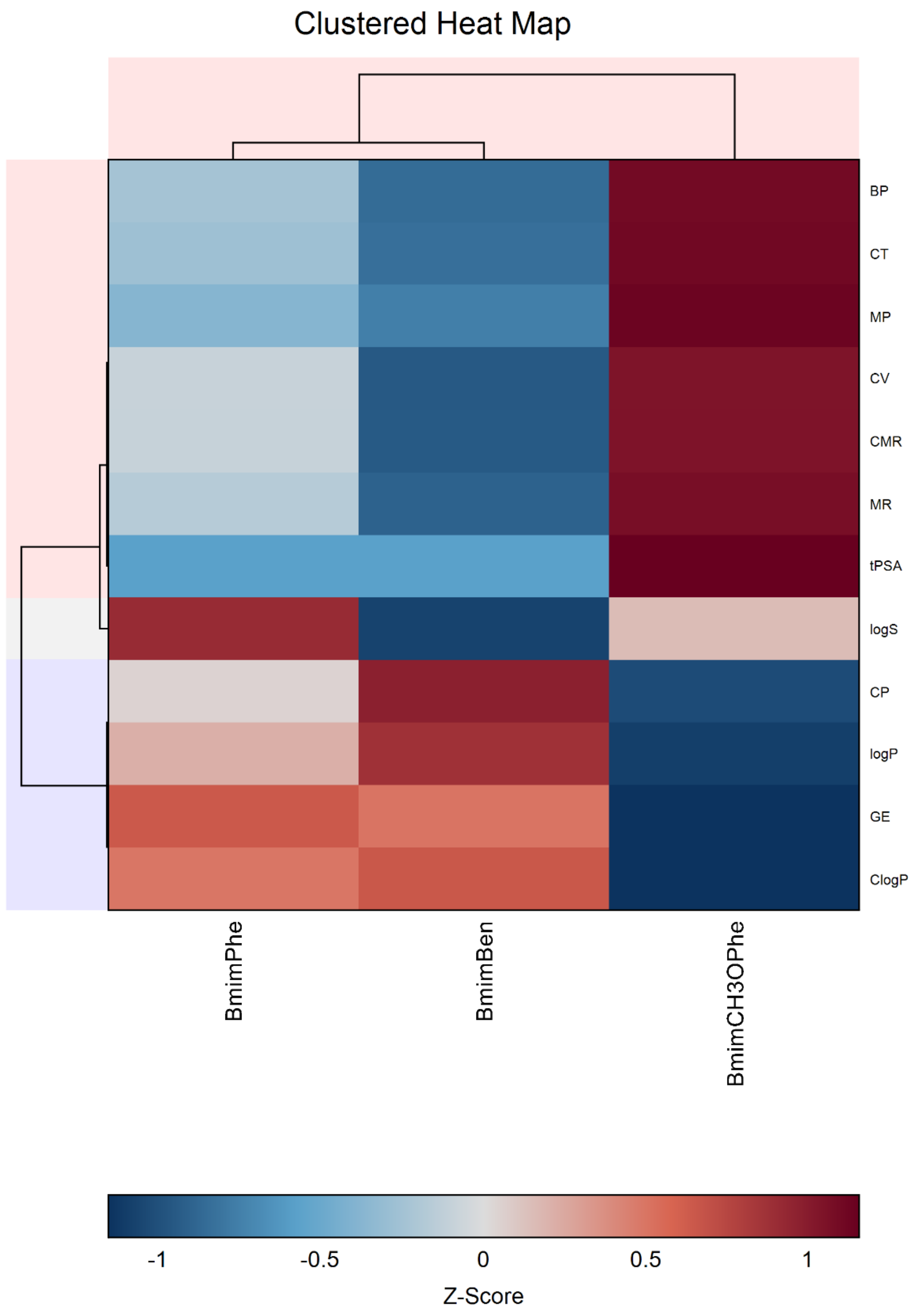

Figure S8. The results of HCA of ILs based on scaled data using Z-scores of a) density, b) viscosity, c) electrical conductivity, d) molar conductivity, e) thermal expansion coefficient, and f) *in silico* physicochemical properties.
